# Geographic and Temporal Trends in the Molecular Epidemiology and Genetic Mechanisms of Transmitted HIV-1 Drug Resistance: An Individual-Patient- and Sequence-Level Meta-Analysis

**DOI:** 10.1371/journal.pmed.1001810

**Published:** 2015-04-07

**Authors:** Soo-Yon Rhee, Jose Luis Blanco, Michael R. Jordan, Jonathan Taylor, Philippe Lemey, Vici Varghese, Raph L. Hamers, Silvia Bertagnolio, Tobias F. Rinke de Wit, Avelin F. Aghokeng, Jan Albert, Radko Avi, Santiago Avila-Rios, Pascal O. Bessong, James I. Brooks, Charles A. B. Boucher, Zabrina L. Brumme, Michael P. Busch, Hermann Bussmann, Marie-Laure Chaix, Bum Sik Chin, Toni T. D’Aquin, Cillian F. De Gascun, Anne Derache, Diane Descamps, Alaka K. Deshpande, Cyrille F. Djoko, Susan H. Eshleman, Herve Fleury, Pierre Frange, Seiichiro Fujisaki, P. Richard Harrigan, Junko Hattori, Africa Holguin, Gillian M. Hunt, Hiroshi Ichimura, Pontiano Kaleebu, David Katzenstein, Sasisopin Kiertiburanakul, Jerome H. Kim, Sung Soon Kim, Yanpeng Li, Irja Lutsar, Lynn Morris, Nicaise Ndembi, Kee Peng NG, Ramesh S. Paranjape, Martine Peeters, Mario Poljak, Matt A. Price, Manon L. Ragonnet-Cronin, Gustavo Reyes-Terán, Morgane Rolland, Sunee Sirivichayakul, Davey M. Smith, Marcelo A. Soares, Vincent V. Soriano, Deogratius Ssemwanga, Maja Stanojevic, Mariane A. Stefani, Wataru Sugiura, Somnuek Sungkanuparph, Amilcar Tanuri, Kok Keng Tee, Hong-Ha M. Truong, David A. M. C. van de Vijver, Nicole Vidal, Chunfu Yang, Rongge Yang, Gonzalo Yebra, John P. A. Ioannidis, Anne-Mieke Vandamme, Robert W. Shafer

**Affiliations:** 1 Department of Medicine, Stanford University, Stanford, California, United States of America; 2 Hospital Clinic Universitari–Institut d’Investigacions Biomèdiques August Pi i Sunyer, University of Barcelona, Barcelona, Spain; 3 Tufts University School of Medicine, Boston, Massachusetts, United States of America; 4 Department of Statistics, Stanford University, Stanford, California, United States of America; 5 KU Leuven—University of Leuven, Department of Microbiology and Immunology, Rega Institute for Medical Research, Clinical and Epidemiological Virology, Leuven, Belgium; 6 Department of Global Health and Internal Medicine, Academic Medical Center of the University of Amsterdam, and Amsterdam Institute for Global Health and Development, Amsterdam, the Netherlands; 7 World Health Organization, Geneva, Switzerland; 8 Virology Laboratory CREMER-IMPM, Yaoundé, Cameroon; 9 Department of Microbiology, Tumor and Cell Biology, Karolinska Institutet, Stockholm, Sweden; 10 Department of Clinical Microbiology, Karolinska University Hospital, Stockholm, Sweden; 11 Department of Microbiology, University of Tartu, Tartu, Estonia; 12 National Institute of Respiratory Diseases, Centre for Research in Infectious Diseases, Mexico City, Mexico; 13 HIV/AIDS & Global Health Research Programme, Department of Microbiology, University of Venda, Thohoyandou, South Africa; 14 National HIV and Retrovirology Laboratories, Public Health Agency of Canada, Ottawa, Ontario, Canada; 15 Department of Viroscience, Erasmus Medical Centre, Erasmus University, Rotterdam, Netherlands; 16 British Columbia Centre for Excellence in HIV/AIDS, Vancouver, British Columbia, Canada; 17 Faculty of Health Sciences, Simon Fraser University, Burnaby, British Columbia, Canada; 18 Blood Systems Research Institute, San Francisco, California, United States of America; 19 Botswana–Harvard AIDS Institute Partnership, Gaborone, Botswana; 20 Laboratoire de Virologie, Hôpital Saint Louis, Université Paris Diderot, INSERM U941, Paris, France; 21 Center for Infectious Diseases, National Medical Center, Seoul, Republic of Korea; 22 CIRBA-Programme PACCI, Abidjan, Côte d’Ivoire; 23 UCD National Virus Reference Laboratory, University College Dublin, Dublin, Ireland; 24 Department of Virology, Pitie-Salpetriere Hospital, Paris, France; 25 Laboratoire de Virologie, Assistance Publique–Hôpitaux de Paris Hôpital Bichat-Claude Bernard, INSERM UMR 1137, Université Paris Diderot, Paris, France; 26 Department of Medicine, Grant Medical College and Sir Jamshedjee Jeejeebhoy Group of Hospitals, Mumbai, India; 27 Global Viral Cameroon, Intendance Round About, EMAT/CRESAR, Yaoundé, Cameroon; 28 Department of Pathology, Johns Hopkins University School of Medicine, Baltimore, Maryland, United States of America; 29 Laboratoire de Virologie, Centre Hospitalier Universitaire de Bordeaux, CNRS UMR 5234, Université de Bordeaux, Bordeaux, France; 30 Microbiology Department, Hôpital Necker-Enfants Malades, Paris, France; 31 Influenza Virus Research Center, National Institute of Infectious Diseases, Tokyo, Japan; 32 National Hospital Organization Nagoya Medical Center, Nagoya, Japan; 33 Department of Microbiology, Hospital Universitario Ramón y Cajal, Instituto Ramón y Cajal de Investigación Sanitaria, Madrid, Spain; 34 Centre for HIV and STIs, National Institute for Communicable Diseases, Johannesburg, South Africa; 35 Department of Viral Infection and International Health, Graduate School of Medical Sciences, Kanazawa University, Kanazawa, Japan; 36 MRC/UVRI Uganda Research Unit on AIDS, Entebbe, Uganda; 37 Faculty of Medicine Ramathibodi Hospital, Mahidol University, Bangkok, Thailand; 38 US Military HIV Research Program, Walter Reed Army Institute of Research, Silver Spring, Maryland, United States of America; 39 Division of AIDS, Korea National Institute of Health, Osong, Chungcheongbuk-do, Republic of Korea; 40 State Key Laboratory of Virology, Wuhan Institute of Virology, Chinese Academy of Sciences, Wuhan, China; 41 Institute of Human Virology, Abuja, Nigeria; 42 Department of Medicine, Faculty of Medicine, University of Malaya, Kuala Lumpur, Malaysia; 43 National AIDS Research Institute, Indian Council of Medical Research, Pune, India; 44 Unité Mixte Internationale 233, Institut de Recherche pour le Développement, INSERM U1175, and University of Montpellier, 34394 Montpellier, France; 45 Computational Biology Institute, Montpellier, France; 46 Institute of Microbiology, Faculty of Medicine, University of Ljubljana, Ljubljana, Slovenia; 47 Department of Medical Affairs, International AIDS Vaccine Initiative, New York, New York, United States of America; 48 Department of Epidemiology and Biostatistics, School of Medicine, University of California, San Francisco, California, United States of America; 49 University of Edinburgh, Edinburgh, Scotland, United Kingdom; 50 Faculty of Medicine, Chulalongkorn University, Bangkok, Thailand; 51 University of California San Diego, La Jolla, California, United States of America; 52 Universidade Federal do Rio de Janeiro, Rio de Janeiro, Brazil; 53 Department of Infectious Diseases, Hospital Carlos III, Madrid, Spain; 54 Institute of Microbiology and Immunology, Faculty of Medicine, University of Belgrade, Belgrade, Serbia; 55 Federal University of Goias, Goias, Brazil; 56 Department of Medicine, University of California, San Francisco, California, United States of America; 57 Institut de Recherche pour le Développement, University of Montpellier 1, Montpellier, France; 58 International Laboratory Branch, Division of Global HIV/AIDS, Center for Global Health, Centers for Disease Control and Prevention, Atlanta, Georgia, United States of America; 59 Stanford Prevention Research Center, Department of Medicine, Stanford University, Stanford, California, United States of America; 60 Meta-Research Innovation Center at Stanford, Stanford University, Stanford, California, United States of America; 61 Global Health and Tropical Medicine, Unidade de Microbiologia, Instituto de Higiene e Medicina Tropical, Universidade Nova de Lisboa, Lisbon, Portugal; St. Vincent's Hospital, AUSTRALIA

## Abstract

**Background:**

Regional and subtype-specific mutational patterns of HIV-1 transmitted drug resistance (TDR) are essential for informing first-line antiretroviral (ARV) therapy guidelines and designing diagnostic assays for use in regions where standard genotypic resistance testing is not affordable. We sought to understand the molecular epidemiology of TDR and to identify the HIV-1 drug-resistance mutations responsible for TDR in different regions and virus subtypes.

**Methods and Findings:**

We reviewed all GenBank submissions of HIV-1 reverse transcriptase sequences with or without protease and identified 287 studies published between March 1, 2000, and December 31, 2013, with more than 25 recently or chronically infected ARV-naïve individuals. These studies comprised 50,870 individuals from 111 countries. Each set of study sequences was analyzed for phylogenetic clustering and the presence of 93 surveillance drug-resistance mutations (SDRMs). The median overall TDR prevalence in sub-Saharan Africa (SSA), south/southeast Asia (SSEA), upper-income Asian countries, Latin America/Caribbean, Europe, and North America was 2.8%, 2.9%, 5.6%, 7.6%, 9.4%, and 11.5%, respectively. In SSA, there was a yearly 1.09-fold (95% CI: 1.05–1.14) increase in odds of TDR since national ARV scale-up attributable to an increase in non-nucleoside reverse transcriptase inhibitor (NNRTI) resistance. The odds of NNRTI-associated TDR also increased in Latin America/Caribbean (odds ratio [OR] = 1.16; 95% CI: 1.06–1.25), North America (OR = 1.19; 95% CI: 1.12–1.26), Europe (OR = 1.07; 95% CI: 1.01–1.13), and upper-income Asian countries (OR = 1.33; 95% CI: 1.12–1.55). In SSEA, there was no significant change in the odds of TDR since national ARV scale-up (OR = 0.97; 95% CI: 0.92–1.02). An analysis limited to sequences with mixtures at less than 0.5% of their nucleotide positions—a proxy for recent infection—yielded trends comparable to those obtained using the complete dataset. Four NNRTI SDRMs—K101E, K103N, Y181C, and G190A—accounted for >80% of NNRTI-associated TDR in all regions and subtypes. Sixteen nucleoside reverse transcriptase inhibitor (NRTI) SDRMs accounted for >69% of NRTI-associated TDR in all regions and subtypes. In SSA and SSEA, 89% of NNRTI SDRMs were associated with high-level resistance to nevirapine or efavirenz, whereas only 27% of NRTI SDRMs were associated with high-level resistance to zidovudine, lamivudine, tenofovir, or abacavir. Of 763 viruses with TDR in SSA and SSEA, 725 (95%) were genetically dissimilar; 38 (5%) formed 19 sequence pairs. Inherent limitations of this study are that some cohorts may not represent the broader regional population and that studies were heterogeneous with respect to duration of infection prior to sampling.

**Conclusions:**

Most TDR strains in SSA and SSEA arose independently, suggesting that ARV regimens with a high genetic barrier to resistance combined with improved patient adherence may mitigate TDR increases by reducing the generation of new ARV-resistant strains. A small number of NNRTI-resistance mutations were responsible for most cases of high-level resistance, suggesting that inexpensive point-mutation assays to detect these mutations may be useful for pre-therapy screening in regions with high levels of TDR. In the context of a public health approach to ARV therapy, a reliable point-of-care genotypic resistance test could identify which patients should receive standard first-line therapy and which should receive a protease-inhibitor-containing regimen.

## Abstract 2

In this individual patient and sequence-level meta-analysis, Soo-Yon Rhee and colleagues measure regional trends in HIV-1 transmitted drug resistance prevalence and investigate the specific mutations responsible for TDR in different regions and in different virus subtypes.

## Editors' Summary

**Background:**

About 35 million people are currently infected with HIV, the virus that causes AIDS by destroying immune system cells and leaving infected individuals susceptible to other infections. Early in the AIDS epidemic, most HIV-infected individuals died within ten years of infection. Then, in 1996, effective antiretroviral (ARV) therapy—drug combinations that suppress HIV replication by inhibiting reverse transcriptase and other essential viral enzymes—became available. For people living in affluent countries, HIV/AIDS became a chronic condition, but because ARV therapy was expensive, HIV/AIDS remained fatal in low- and middle-income countries (LMICs). In 2003, the international community began to work towards achieving universal access to ARV therapy. Now, more than 10 million HIV-positive individuals in LMICs receive ARV therapy, usually as a fixed-dose combination of two nucleoside reverse transcriptase inhibitors (NRTIs), such as tenofovir and lamivudine, plus a non-nucleoside reverse transcriptase inhibitor (NNRTI), such as efavirenz or nevirapine.

**Why Was This Study Done?:**

The global scale-up of ARV therapy has reduced deaths from HIV/AIDS and the incidence of HIV infection in LMICs, but the development of resistance to ARV therapy is threatening these advances. HIV rapidly accumulates genetic changes (mutations), some of which make HIV resistant to ARV therapy. Up to 30% of patients receiving a fixed-dose NRTI/NNRTI combination develop virological failure, and a high proportion of these patients develop mutations associated with resistance to the ARVs in their regimen. Moreover, the proportion of newly infected, ARV-naïve individuals with transmitted drug resistance (TDR) is also increasing. Organizations involved in HIV/AIDS control need to understand the regional and temporal mutational patterns of TDR to inform the development of guidelines for first-line ARV therapy and of inexpensive resistance mutation assays for use in LMICs. Here, using a statistical approach called meta-analysis to combine information from individual patients about the resistance mutations they carry, the researchers investigate the molecular epidemiology of TDR (the patterns of molecular changes underlying TDR in populations) and identify the HIV drug-resistance mutations most responsible for TDR in different world regions.

**What Did the Researchers Do and Find?:**

The researchers identified 287 studies published between 2000 and 2013 from 111 countries that included the reverse transcriptase sequences of HIV viruses from 50,870 ARV-naïve, HIV-positive individuals. The researchers analyzed each virus sequence for the presence of 93 surveillance drug-resistance mutations (SDRMs) previously shown to be specific indicators of TDR. Meta-analysis of these data indicated that the average overall prevalence of TDR (the proportion of ARV-naïve, HIV-positive individuals infected with a virus carrying one or more SDRMs) ranged from 2.8% in sub-Saharan Africa to 11.5% in North America. In sub-Saharan Africa, the odds (chance) of TDR increased 1.09-fold per year following national ARV scale-up; this increase was attributable to an increase in NRTI- and NNRTI-associated resistance. By contrast, in LMICs in south/southeast Asia, the odds of TDR remained unchanged following ARV scale-up. In Latin America/Caribbean, North America, Europe, and upper-income Asian countries, the odds of TDR have increased by around 1.10-fold per year since 1995, mainly as a result of increased NNRTI resistance. Four NNRTI-associated and 16 NRTI-associated SDRMs accounted for most NNRTI- and NRTI-associated TDR, respectively, in all regions. Notably, in sub-Saharan Africa and south/southeast Asia, most of the NNRTI-associated SDRMs detected were associated with high-level resistance to nevirapine or efavirenz. Finally, the researchers report that 95% of TDR viruses in sub-Saharan Africa and south/southeast Asia were unrelated and had therefore arisen independently.

**What Do These Findings Mean?:**

Because many drug-resistance mutations reduce HIV’s fitness and tend to be lost rapidly in individuals not exposed to ARV therapy, differences among the datasets used in this meta-analysis with respect to how long each ARV-naïve patient had been infected with HIV before virus sampling may limit the accuracy of these findings. Nevertheless, the finding that most of the TDR strains detected in sub-Saharan Africa and south/southeast Asia arose independently suggests that improved patient adherence to ARV therapy and the use of ARV regimens that contain drugs to which HIV rarely develops resistance (regimens with a high genetic barrier to resistance) should reduce the generation of new ARV-resistant strains and mitigate TDR increases. In addition, the finding that a few NNRTI-resistance mutations were responsible for most cases of transmitted high-level resistance suggests that an inexpensive assay that detects these specific mutations may be useful for pre-therapy screening in LMICs with high TDR levels.

**Additional Information:**

Please access these websites via the online version of this summary at http://dx.doi.org/10.1371/journal.pmed.1001810.

- Information is available from the US National Institute of Allergy and Infectious Diseases on HIV infection and AIDS
- NAM/aidsmap provides basic information about HIV/AIDS, summaries of recent research findings on HIV care and treatment, and personal stories about living with HIV/AIDS
- Information is available from Avert, an international AIDS charity, on many aspects of HIV/AIDS, including information on antiretroviral drugs and on universal access to ARV therapy; Avert also provides personal stories about living with HIV/AIDS
- The World Health Organization provides information on all aspects of HIV/AIDS (in several languages), including its guidelines on the use of antiretroviral therapy for treating and preventing HIV infection
- The UNAIDS *World AIDS Day Report 2014* provides up-to-date information about the AIDS epidemic and efforts to halt it, including progress towards universal access to antiretroviral therapy
- The Stanford University HIV Drug Resistance Database includes information about surveillance drug-resistant mutations (SDRMs) and an interactive map displaying HIV drug resistance in ARV-naïve populations

## Introduction

More than 10 million individuals in low- and middle-income countries (LMICs) are receiving antiretroviral (ARV) therapy [1]. The global scale-up of ARV therapy has markedly reduced HIV-1 mortality, mother-to-child transmission, and adult HIV-1 incidence [2–5]. These unprecedented public health accomplishments were made possible by the availability and widespread administration of inexpensive fixed-dose combinations of two nucleoside reverse transcriptase inhibitors (NRTIs) plus a non-nucleoside reverse transcriptase inhibitor (NNRTI) [6,7].

However, the margin of long-term ARV treatment success in LMICs is narrow because NNRTI-based regimens have a low genetic barrier to resistance. ARV treatment failure with a fixed-dose NRTI/NNRTI combination occurs in 10% to 30% of patients per year [8–10], and most patients with virological failure acquire NRTI and/or NNRTI resistance [10–12]. As the number of LMIC patients with acquired ARV resistance has increased, so has the proportion of newly infected patients with transmitted drug resistance (TDR) [11,13,14].

Although both acquired and transmitted HIV-1 drug resistance are public health concerns, TDR has the potential to more rapidly reverse the effectiveness of first-line ARV therapy at the population level. Persons with TDR who begin ARV therapy with a lower genetic barrier to resistance have a higher risk of virological failure [15–20]. Previous meta-analyses have examined aggregate data from studies of TDR in different regions at different times but have not examined the virus sequences responsible for TDR. In this study, we performed an individual-patient-level meta-analysis to characterize the molecular epidemiology of transmitted HIV-1 drug-resistant variants and to identify the drug-resistance mutations most responsible for TDR in different regions and virus subtypes.

## Methods

### Study Inclusion Criteria

We retrieved all published HIV-1 group M reverse transcriptase (RT) nucleic acid sequences, with or without protease sequences, using a tblastn search of the GenBank nucleotide sequence database v. 200 (released 2014-02-15). Retrieved sequences with the same GenBank “Author” and “Title” fields were grouped into submission sets (or studies). We then read the GenBank annotation and associated published papers to identify studies meeting the following two criteria: (i) studies that described a population of ≥25 ARV-naïve HIV-1-infected individuals characterized by country, year of virus sampling, and method and site of recruitment, and (ii) studies that contained sequences encompassing RT codons 40 to 240 determined by direct PCR sequencing of plasma, peripheral blood mononuclear cells, or dried blood spots. Studies of unrepresentative populations, such as those in which individuals were selected based on knowledge of their ARV-resistance status, were excluded. Studies of children born to mothers receiving ARV therapy were also excluded.

Studies meeting inclusion criteria were assigned to one of the following geo-economic regions: (i) sub-Saharan Africa (SSA), (ii) LMICs of south/southeast Asia (SSEA), (iii) Latin America and Caribbean, (iv) Europe, (v) United States, Canada, and Puerto Rico (North America), (vi) upper-income Asian countries, (vii) countries of the former Soviet Union (FSU), (viii) North Africa, and (ix) Australia. For studies conducted in countries on different continents, separate datasets for each continent were created, provided the study had more than 25 individuals per country.

### Sequence Analyses

TDR was defined as the presence in ARV-naïve individuals of one or more mutations from the WHO 2009 list of surveillance drug-resistance mutations (SDRMs) [21]. The SDRM list consists of 93 drug-resistance mutations, including 34 NRTI-resistance mutations at 15 RT positions, 19 NNRTI-resistance mutations at ten RT positions, and 40 protease inhibitor (PI)–resistance mutations at 18 protease positions. Thymidine-analog mutations (TAMs) were defined as the NRTI SDRMs M41L, D67N/G/E, K70R, L210W, T215Y/F/S/C/D/E/I/V, and K219Q/E/N/R. T215 mutations other than T215Y/F were called T215 revertants because they often emerge in individuals initially infected with a virus containing T215Y/F [22,23].

The Calibrated Population Resistance (CPR) analysis tool (http://cpr.stanford.edu/cpr.cgi) was used to calculate the proportions of individuals per study with overall and NRTI-, NNRTI-, and PI-associated TDR [24]. CPR was also used for quality control, excluding sequences containing an excess of stop codons, highly ambiguous nucleotide calls, extensive G-to-A hypermutation, or highly unusual amino acids. HIV-1 subtype was determined using the REGA HIV-1 Subtyping Tool [25].

We also determined the proportion of bases containing electrophoretic evidence for a mixture of two nucleotides. We then examined whether the median proportion of mixtures in a study correlated with characteristics of the study population such as whether the study population comprised individuals known to belong to groups likely to be recently infected, such as primiparous women presenting for antenatal care. In subset analyses designed to include individuals more likely to have been recently infected, samples were classified as having a low (<0.5%) or high (≥0.5%) proportion of mixtures based on previous studies showing that a 0.5% cutoff is useful for identifying recently infected individuals [26–28].

### Temporal Changes in Prevalence of Transmitted Drug Resistance

For individual-patient-level meta-analyses, samples from different studies conducted in the same region were pooled and a generalized linear mixed model was used to assess the effects of national ARV scale-up on the presence or absence of any or NRTI-, NNRTI-, or PI-associated TDR [29]. To account for study heterogeneity, we included study as a random effect in the model using the R package lme4 [30]. We calculated the yearly increase in the odds of TDR since ARV scale-up. We also assessed the associations of virus subtype, duration of HIV-1 infection, recruitment site, and sample type with the odds of TDR while accounting for the number of years since national ARV scale-up. The year of each country’s national ARV scale-up was obtained from UN General Assembly special session country reports. For regions other than SSA and SSEA, we used sample year (rather than years since ARV scale-up) in the generalized linear mixed model because in these regions ARVs were more often available to the general population in the 1990s.

We also performed two subset analyses to assess the robustness of the overall model to two sources of potential variation: the duration of infection prior to virus sequencing and the nature of patient recruitment. In the first subset analysis, we performed generalized linear mixed regression using only virus sequences with mixtures at less than 0.5% of their nucleotide positions—a proxy for recent infection. In the second subset analysis, we performed generalized linear mixed regression using the subset of studies in which participants were sequentially recruited.

### Mutation Analyses

We compared the proportions of each SDRM in sequences from the seven most common HIV-1 subtypes (subtypes A, B, C, D, G, CRF01_AE, and CRF02_AG) and from individuals from SSA, SSEA, Latin America/Caribbean, and the pooled upper-income countries of North America, Europe, and Asia using Fisher’s exact test. Holm’s method was used to control the family-wise error rate for multiple hypothesis testing: associations with adjusted *p* < 0.05 were considered statistically significant. Statistically significant associations are reported, along with their original unadjusted *p-*values.

We used Spearman’s rank correlation test to assess the correlation of the relative ranking of the proportions of each NRTI, NNRTI, and PI SDRM in SSA, SSEA, Latin America/Caribbean, and the pooled upper-income countries with the proportions of these mutations in HIV-1 sequences from ARV-experienced individuals from these regions in the Stanford University HIV Drug Resistance Database (HIVDB). For this analysis, we excluded all sequences from studies of ARV-experienced individuals selected on the basis of their patterns of drug-resistance mutations. The numbers of included NRTI-treated individuals were 4,522 (SSA), 2,218 (SSEA), 4,164 (Latin America/Caribbean), and 13,522 (pooled upper-income countries). The numbers of NNRTI-treated individuals were 4,959 (SSA), 1,994 (SSEA), 3,677 (Latin America/Caribbean), and 8,927 (pooled upper-income countries). The numbers of PI-treated individuals were 717 (SSA), 103 (SSEA), 4,107 (Latin America/Caribbean), and 9,985 (pooled upper-income countries). We also analyzed the correlation between the presence of an SDRM in a sequence and the estimated level of drug resistance for that sequence according to the HIVDB genotypic resistance interpretation system [31].

### Molecular Epidemiology

A neighbor-joining tree of each study’s sequences was created using genetic distances computed using the HKY85 substitution model with a gamma distribution to model site rate variation. By traversing the tree, we identified sets of closely related sequences for which the median genetic distance was ≤0.015. An SDRM cluster was defined as a set of three or more closely related sequences containing identical SDRMs. Trees were constructed using PAUP and traversed using R packages ape and igraph [32]. The program BEAST (Bayesian Evolutionary Analysis by Sampling Trees) was used to identify extended lineages of sequence clusters with the same SDRMs [33].

For each study, we calculated a sequence dissimilarity index, which we defined as the number of sequence clusters plus unclustered sequences divided by the total number of sequences. Using this approach, studies without any closely related sequences had a sequence dissimilarity index of 100%. To assess the impact of closely related sequences on the proportion of individuals with TDR, we recalculated this proportion counting closely related sequences with identical SDRMs just once, assuming these reflected transmission of resistant viruses among ARV-naïve patients. We then recalculated the TDR prevalence in each study to yield an estimate reflecting transmission from ARV-treated to ARV-naïve individuals.

## Results

### Studies and Individuals

**Fig 1:**
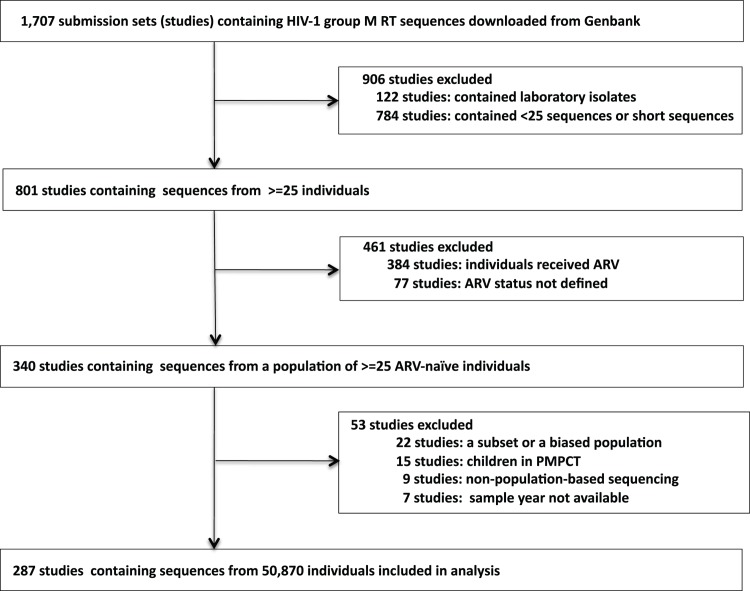
Flow chart showing the derivation of study sets meeting meta-analysis inclusion criteria: studies of representative ARV-naïve populations of 25 or more individuals with published RT sequences with or without protease sequences.

The February 2014 GenBank tblastn search yielded 1,707 studies of HIV-1 group M RT sequences, with or without protease sequences. Of these studies, 340 described a population of ≥25 ARV-naïve individuals. Fifty-three of the 340 studies were excluded: 22 described an unrepresentative subset of a larger population, 15 included children in a program for prevention of mother-to-child transmission, nine included samples sequenced using a method other than direct PCR sequencing, and seven did not include sample years. Additionally, 111 individuals were excluded because their sequences did not meet sequence quality inclusion criteria. Finally, sequences from 50,870 ARV-naïve individuals in 287 studies were included in our analysis (Fig 1; S1 Table).

For 277 (97%) studies, annotation was obtained from an accompanying peer-reviewed publication. For ten (3%) studies, annotation was obtained from the GenBank record and the sequence contributors. The primary goal for 221 (77%) studies was to estimate TDR prevalence. The primary goal for 62 (22%) was to characterize sequence diversity for molecular epidemiologic purposes or vaccine development. Four (1%) studies contained pre-therapy samples from patients enrolling in a clinical trial.

For 238 (83%) of the studies, the sample year of each sequence was reported. For 49 (17%) of the studies, a range of sample years was reported for the study population rather than for each individual, and the median of the range was assigned to each sample. Sequences were obtained from plasma, peripheral blood mononuclear cells, and dried blood spots in 252 (88%), 29 (10%), and six (2%) studies, respectively. Both RT and protease were sequenced in 272 (95%) studies; only RT was sequenced in 15 (5%) studies.

In 211 (73.5%) studies, cohorts were composed of sequentially recruited individuals characterized by region, time period, and site of recruitment. In 21 (7.3%) studies, cohorts were a random subset of sequentially recruited individuals characterized by region, time period, and site of recruitment. Thus, overall, participants from 232 (80.8%) studies were sequentially recruited. In 47 (16.4%) studies, participants were not sequentially recruited but rather were an unbiased subset of available samples from individuals characterized by region, time period, and site of recruitment. In six (2.1%) studies, the method of participant recruitment was not provided. In two (0.7%) studies, participants were recruited using respondent-driven sampling.

### ARV-Naïve Population Characteristics by Region

**Fig 2:**
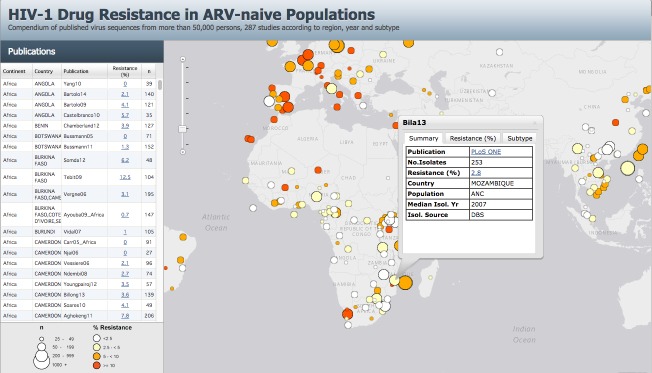
A snapshot of an interactive map plotting the prevalence of transmitted drug resistance in 111 countries from 287 studies between 2000 and 2013 (http://hivdb.stanford.edu/surveillance/map/). Each study is represented by a circle. The size of the circle is proportional to the number of individuals in the study. The circle color indicates the prevalence of overall TDR in the study: white (<2.5%), pale yellow (2.5% to 4.9%), orange (5.0% to 9.9%), and red (≥10.0%). Each study can also be located on a sidebar, which lists each publication, percent overall TDR, number of individuals, and the country (or countries) where the study was conducted. Clicking on a sidebar row or a study circle in the interactive version of the map at http://hivdb.stanford.edu/surveillance/map/ generates a pop-up box with additional information including a link to the appropriate PubMed reference, the TDR prevalence by ARV class, the median year of virus sampling, the source of virus isolation, the mechanism of participant recruitment, and the virus subtype distribution (a pop-up box of the study Bila13 is shown as an example). The complete set of data associated with a study can be reviewed by clicking on the “Resistance (%)” link either on the sidebar or within the study circle pop-up menu.

There was a median of 91 individuals per study (interquartile range [IQR]: 49–174). Ninety-five (33%) of 287 studies were conducted in SSA (11,536 individuals; 32 countries), 56 (20%) in SSEA (6,522 individuals; seven countries), 42 (15%) in Europe (11,802 individuals; 30 countries), 38 (13%) in Latin America/Caribbean (5,628 individuals; 20 countries), 27 (9%) in North America (9,283 individuals; four countries), 12 (4%) in the upper-income countries of Asia (4,950 individuals; five countries), 12 (4%) in FSU countries (1,365 individuals; nine countries), three (1%) in North Africa (157 individuals; three countries), and two (1%) in Australia (627 individuals). Table 1 summarizes the epidemiologic characteristics and virus subtypes, and Table 2 summarizes the median TDR prevalence by ARV class in the seven most commonly studied regions. The epidemiologic characteristics, TDR prevalence, CPR analysis, and link to each study publication can be accessed using an interactive map on the HIVDB website (http://hivdb.stanford.edu/surveillance/map/; Fig 2).

The populations studied in SSA and SSEA were primarily from specialized clinics, including antenatal clinics, voluntary counseling and testing centers, blood donation centers, sexually transmitted diseases clinics, and tuberculosis clinics. The populations studied in Latin America/Caribbean and the upper-income countries were primarily from HIV clinics. Thirty-five (12%) of the 287 studies consisted entirely of individuals with recent HIV-1 infection, including 10% (15) of the 151 studies in SSA and SSEA and 25% (17) of the 69 of studies in Europe and North America. Of the 21 WHO TDR surveillance studies for which sequences were available, 20 were conducted in SSA and SSEA.

**Table 1:** Epidemiologic characteristics in seven geo-economic regions.

Characteristic	SSA	SSEA	Latin America/Caribbean	Europe	North America	Upper-Income Asia	FSU
Number of studies	95	56	38	42	27	12	12
Number of individuals	11,536	6,522	5,628	11,802	9,283	4,950	1,365
Median number individuals per study (IQR)	72 (39–122)	76 (46–123)	82 (50–119)	122 (66–213)	274 (66–675)	339 (68–504)	101 (46–153)
Number of countries	32	7	20	30	4	5	9
Most common countries (number of studies)	ZA (19),UG (13),CM (13)	CN (22),VN (12),IN (11)	BR (24),AR (3),MX (3)	ES (12),IT (7),SE (5)	US (21),CA (9),PR (2)	KR (5),JP (4),TW (2)	EE (3),RU (3),UA (2)
Median sample year (IQR)	2007(2004–2008)	2008(2007–2009)	2007(2002–2008)	2005(2003–2006)	2003(1999–2006)	2005(2004–2007)	2003(2002–2008)
Most common recruitment sites (number of studies)	VCT/ANC/BD/STD/TB (52);HIVC (29)	VCT/ANC/BD (14);HIVC (20)	HIVC (23);VCT/BD (9)	HIVC	HIVC (14);BD (5);VCT (1)	HIVC (8)	VCT/HIVC
Most common virus subtypes (percent individuals)	C (42%),A (17%),02 (17%)	01 (66%),C (15%),B (13%)	B (83%),C (9%)	B (67%),C (7%),G (7%)	B (97%)	B (84%),01 (10%)	A (57%),06 (29%),B (9%)

Latin America/Caribbean includes three studies from Caribbean countries. Three studies from North Africa and two studies from Australia are not included in this table but are summarized in S1 Table. Country abbreviations: AR, Argentina; BR, Brazil; CA, Canada; CM, Cameroon; CN, China; EE, Estonia; ES, Spain; IN, India; IT, Italy; JP, Japan; KR, Republic of Korea; MX, Mexico; PR, Puerto Rico; RU, Russia; SE, Sweden; TW, Taiwan; UA, Ukraine; UG, Uganda; VN, Viet Nam; ZA, South Africa. Recruitment site abbreviations: ANC, antenatal clinics; BD, blood donation centers; HIVC, HIV clinics; STD, sexually transmitted disease clinics; TB, tuberculosis clinics; VCT, voluntary counseling and testing centers.

**Table 2:** Study-level estimates of transmitted drug resistance in seven geo-economic regions.

TDR	SSA (*n* = 95)	SSEA (*n* = 56)	Latin America/Caribbean (*n* = 38)	Europe (*n* = 42)	North America (*n* = 27)	Upper-Income Asia (*n* = 12)	FSU (*n* = 12)
Overall	2.8% (1.3%–5.6%)	2.9% (1.8%–5.3%)	7.6% (3.9%–10.2%)	9.4% (6.1%–15.1%)	11.5% (8.3%–14.6%)	5.6% (3.5%–9.0%)	4.0% (0%–6.4%)
NRTI	0% (0%–2.4%)	1% (0%–2.4%)	4% (1.8%–6.6%)	5.6% (3.1%–10.1%)	5.8% (3.4%–8.2%)	3.5% (1.5%–5.0%)	1.8% (0%–3.9%)
NNRTI	1.4% (0%–2.8%)	0.8% (0%–2.1%)	2.8% (1.1%–5.0%)	3.4% (1.5%–5.3%)	4.5% (3.0%–6.8%)	1.1% (0.2%–1.6%)	0.8% (0%–2.1%)
PI	0% (0%–1.4%)	0.5% (0%–1.9%)	1.4% (0%–3.0%)	1.5% (0%–2.8%)	3.0% (2.3%–3.9%)	1.6% (0.6%–3.0%)	0.2% (0%–2.1%)

Data are median (IQR) of study-level prevalence of individuals with any (overall) and NRTI-, NNRTI-, and PI-associated SDRMs by region; the number of studies conducted is indicated for each region (*n*). Latin America/Caribbean includes three studies from Caribbean countries. Three studies from North Africa and two studies from Australia are not included in this table but are summarized in S1 Table.

The proportion of mixed nucleotide positions per sequence was significantly lower in the samples from the 35 studies consisting entirely of recently infected persons compared with the remaining studies (median 0% versus 0.23% mixtures per sample, *p* < 0.001, Wilcoxon rank sum test). Among these remaining studies, the proportion of mixed nucleotide positions per sequence was significantly lower among blood donors (median 0.08% mixtures per sample), voluntary counseling and testing center attendees (0.22% mixtures per sample), and antenatal clinic attendees (0.28% mixtures per sample) compared with those presenting to an HIV clinic (0.41% mixtures per sample; *p* < 0.001 for each comparison, Wilcoxon rank sum test).

SSA had the most diverse virus subtypes, with C (4,849 viruses; 42%), A (1,991 viruses; 17%), and CRF02_AG (1,982 viruses; 17%) accounting for more than 75% of 11,536 viruses. In SSEA, CRF01_AE (4,270 viruses; 66%), C (1,006 viruses; 15%), and B (856 viruses; 13%) accounted for 95% of 6,522 viruses. In North America, Europe, Latin America/Caribbean, and the upper-income Asian countries, most samples had subtype B viruses (range: 67%–97%). Of 1,365 viruses from FSU countries, the most common subtype was A (783 viruses; 57%).

### Regional Transmitted Drug Resistance Prevalence

The median study-level TDR prevalence ranged from 2.8% and 2.9% in 95 SSA studies and 56 SSEA studies, respectively, to 9.4% and 11.5% in 42 Europe studies and 27 North America studies, respectively (Table 2). Genotypic evidence of two-class TDR was present in 0.6% (69 of 11,536), 0.6% (41 of 6,522), 1.4% (79 of 5,628), and 1.2% (312 of 25,035) of individuals from SSA, SSEA, Latin America/Caribbean, and the pooled upper-income countries, respectively. Genotypic evidence of three-class TDR was present in 0.03% (three of 11,536), 0.04% (three of 6,522), 0.2% (11 of 5,628), and 0.3% (86 of 25,035) of individuals from SSA, SSEA, Latin America and the pooled upper-income countries, respectively.

In 25 of the 95 studies in SSA, most samples were obtained before the national ARV scale-up (median 2 y before scale-up; range: 0–7 y). The median TDR prevalence in these 25 pre-scale-up studies was 2.1% (IQR: 0%–3.3%). In four (15%) of the 25 pre-scale-up studies, TDR prevalence was above 5%. For the remaining 70 post-scale-up studies (median 4 y after scale-up; range: 1–12 y), the median TDR prevalence was 3.2% (IQR: 1.9%–5.7%). In 23 (33%) of the 70 post-scale-up studies, TDR prevalence was above 5%.

In seven of the 56 studies in SSEA, most samples were obtained before the national ARV scale-up (median 2 y before scale-up; range: 0–7 y). The median TDR prevalence in these seven pre-scale-up studies was 2.9% (IQR: 1.0%–5.1%). In two (29%) of the seven studies, TDR prevalence was above 5%. For the remaining 49 post-scale-up studies (median 4 y after scale-up; range: 1–9 y), the median TDR prevalence was 3.0% (IQR: 1.9–5.3%). In 15 (31%) of the 49 post-scale-up studies, TDR prevalence was above 5%.

**Fig 3:**
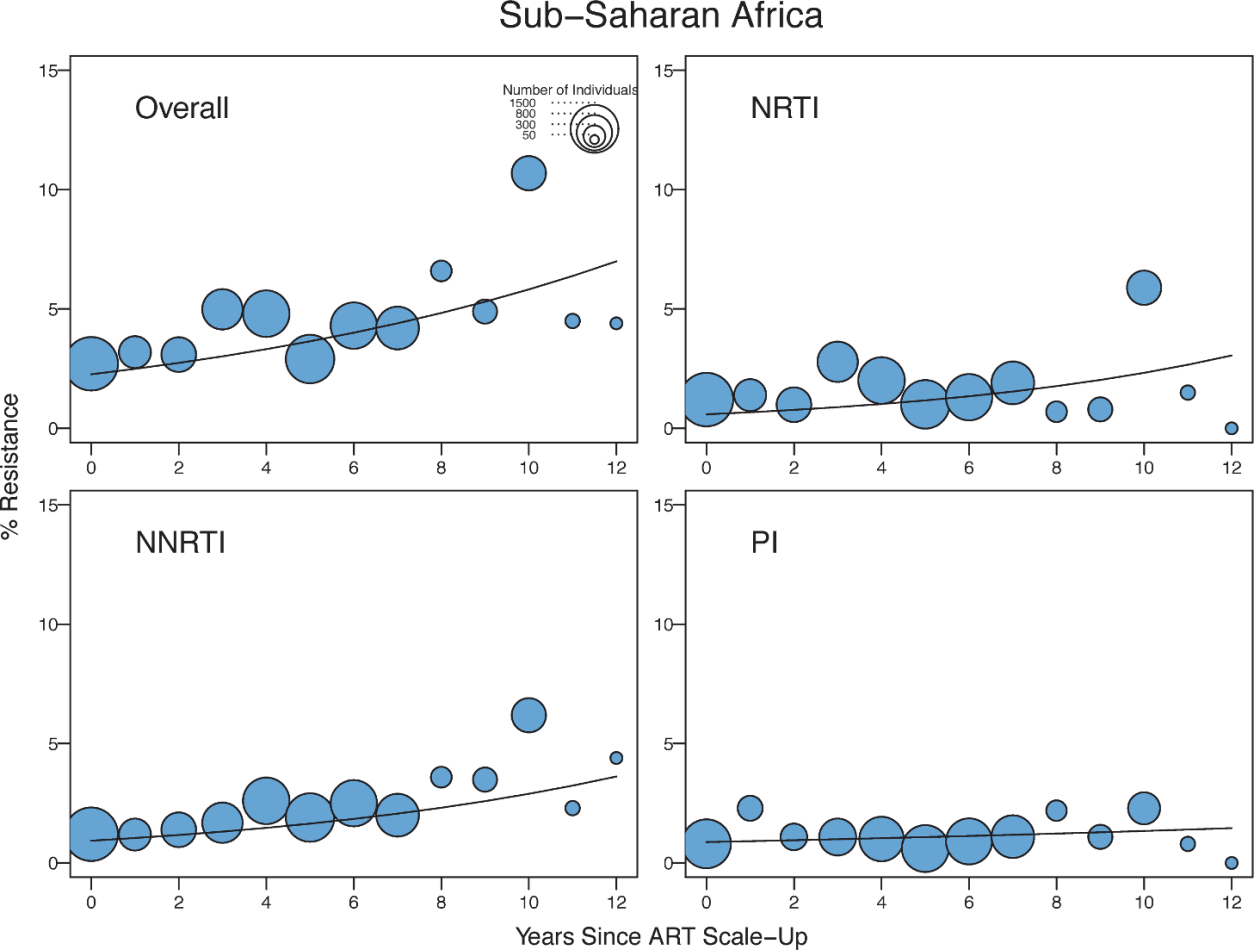
Temporal trends in the yearly proportion of individuals having one or more surveillance drug-resistance mutations in sub-Saharan Africa. The *x*-axes represent the number of years since ARV scale-up for each isolate. The diameter of each circle is proportional to the number of samples sequenced that year. The fitted line shows the fixed effect of years since ARV scale-up in generalized linear mixed model regression.

**Fig 4:**
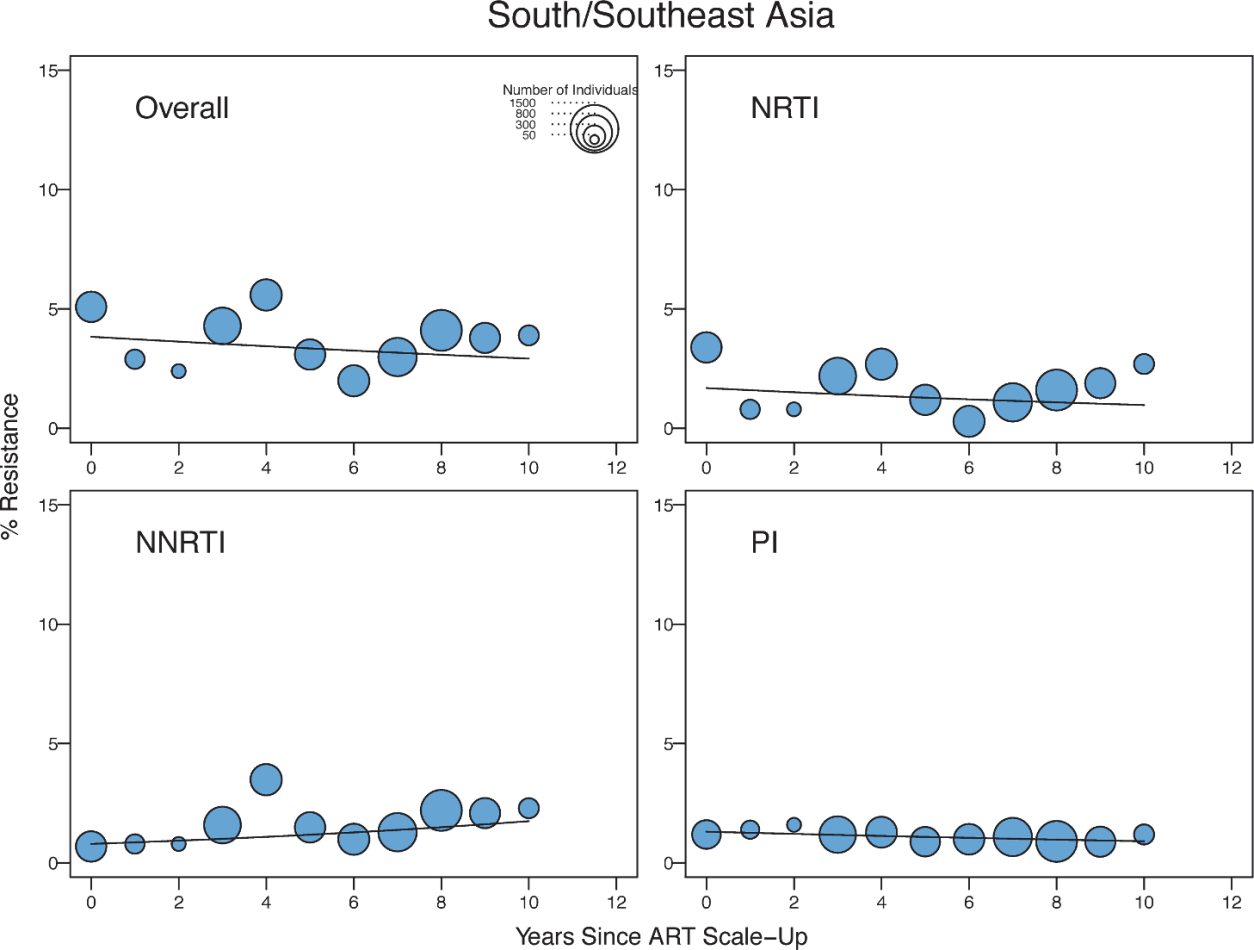
Temporal trends in the yearly proportion of individuals having one or more surveillance drug-resistance mutations in low- and middle-income countries of south and southeast Asia. The *x*-axes represent the number of years since ARV scale-up for each isolate. The diameter of each circle is proportional to the number of samples sequenced that year. The fitted line shows the fixed effect of years since ARV scale-up in generalized linear mixed model regression.

Table 3 shows the odds ratios (ORs) for the yearly change in the proportion of individuals with TDR by general linear mixed regression modeling by year since ARV scale-up in SSA and SSEA and by sample year in the remaining regions. In SSA, there was a significant yearly 1.09-fold (95% CI: 1.05–1.14) increase in the odds of overall TDR, accompanied by an increase in NRTI-associated and NNRTI-associated TDR (Table 3; Fig 3). In SSEA, there was no significant trend over time in overall, NRTI-associated, or NNRTI-associated TDR (Table 3; Fig 4).

**Table 3:** Yearly change in odds of transmitted drug resistance in generalized linear mixed regression models in geo-economic regions with and without ARV scale-up.

Region	Drug Class	OR^a^ (95% CI)	*p*-Value^a^
**OR for years since ARV scale-up** ^**b**^			
SSA(*n =* 11,536)	Overall	1.09 (1.05–1.14)	<0.001
	NRTI	1.12 (1.05–1.19)	<0.001
	NNRTI	1.12 (1.07–1.17)	<0.001
	PI	1.04 (0.99–1.10)	0.1
SSEA(*n =* 6,522)	Overall	0.97 (0.92–1.02)	0.3
	NRTI	0.93 (0.87–1.00)	0.06
	NNRTI	1.09 (0.99–1.21)	0.1
	PI	0.97 (0.90–1.03)	0.4
**OR for sample year** ^**b**^			
Latin America/Caribbean (*n =* 5,628)	Overall	1.06 (1.00–1.11)	0.04
	NRTI	0.99 (0.93–1.07)	0.9
	NNRTI	1.16 (1.06–1.25)	<0.001
	PI	1.01 (0.95–1.07)	0.8
Europe(*n =* 10,802)	Overall	0.97 (0.93–1.00)	0.05
	NRTI	0.93 (0.90–0.93)	<0.001
	NNRTI	1.07 (1.01–1.13)	0.01
	PI	0.99 (0.93–1.05)	0.7
North America(*n =* 9,283)	Overall	1.05 (1.02–1.09)	0.003
	NRTI	1.00 (0.96–1.05)	0.9
	NNRTI	1.19 (1.12–1.26)	<0.001
	PI	1.00 (0.95–1.05)	0.9
Upper-income Asian countries(*n =* 4,950)	Overall	1.15 (1.07–1.23)	<0.001
	NRTI	1.05 (0.96–1.13)	0.3
	NNRTI	1.33 (1.12–1.55)	<0.001
	PI	1.28 (1.12–1.46)	<0.001

Three studies from North Africa and two studies from Australia were excluded. Latin America/Caribbean includes three studies from Caribbean countries.

^a^For each region, a generalized linear mixed model was used to assess the yearly change in the odds (OR) of TDR accounting for study heterogeneity using the R package lme4. The model included a categorical outcome variable indicating the presence or absence of TDR and two explanatory variables: years since scale-up (or the sample year) as a fixed-effect term and the study as a random-effect term.

^b^Yearly change in the odds of TDR since ARV scale-up in regions with national ARV scale-up programs and for each sample year in regions without national ARV scale-up; the number of individuals in each region (*n*) is indicated.

**Fig 5:**
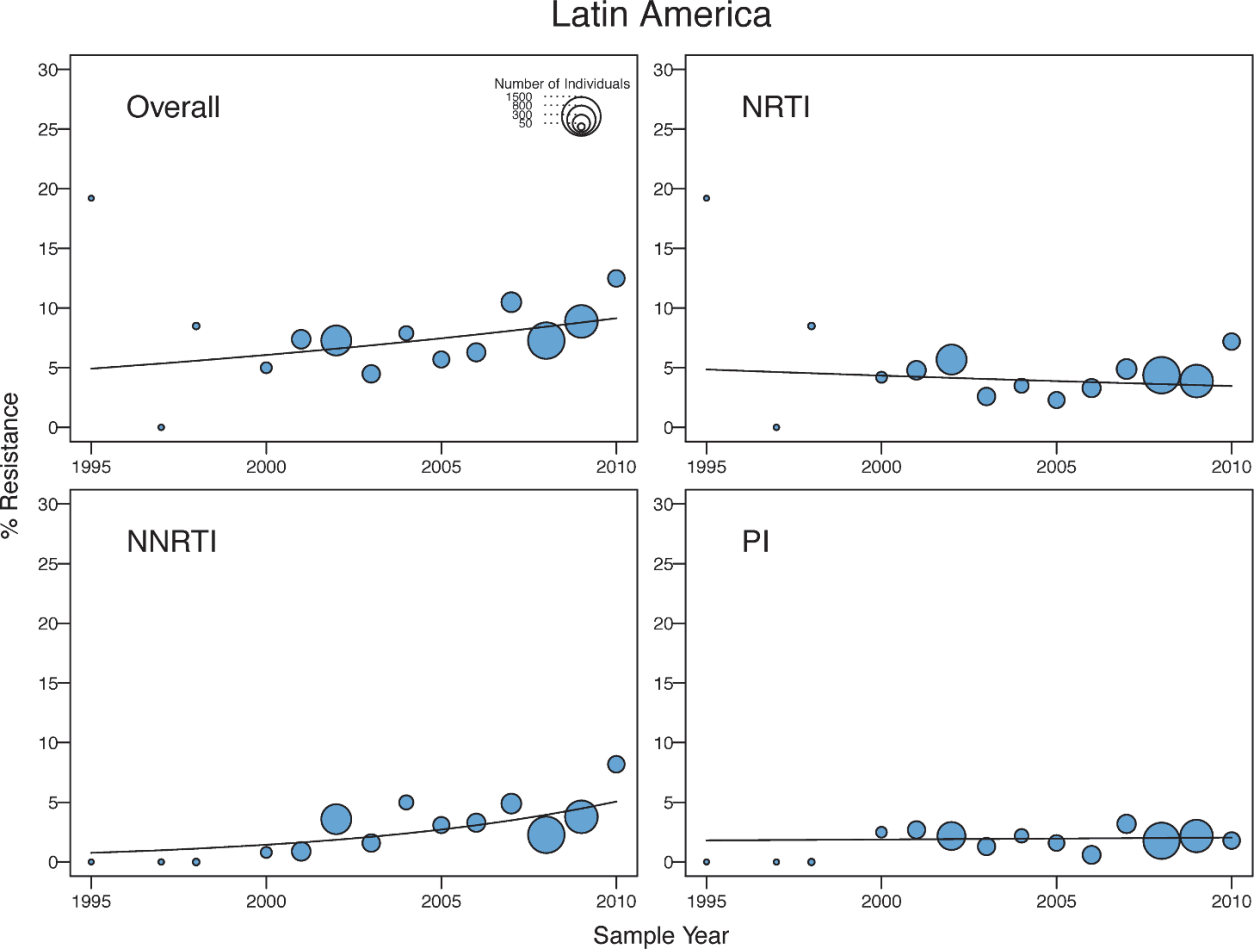
Temporal trends in the yearly proportion of individuals having one or more surveillance drug-resistance mutations in Latin America/Caribbean. The *x*-axes represent the calendar year of the sample. The diameter of each circle is proportional to the number of samples sequenced that year. The fitted line shows the fixed effect of sample year in generalized linear mixed model regression.

**Fig 6:**
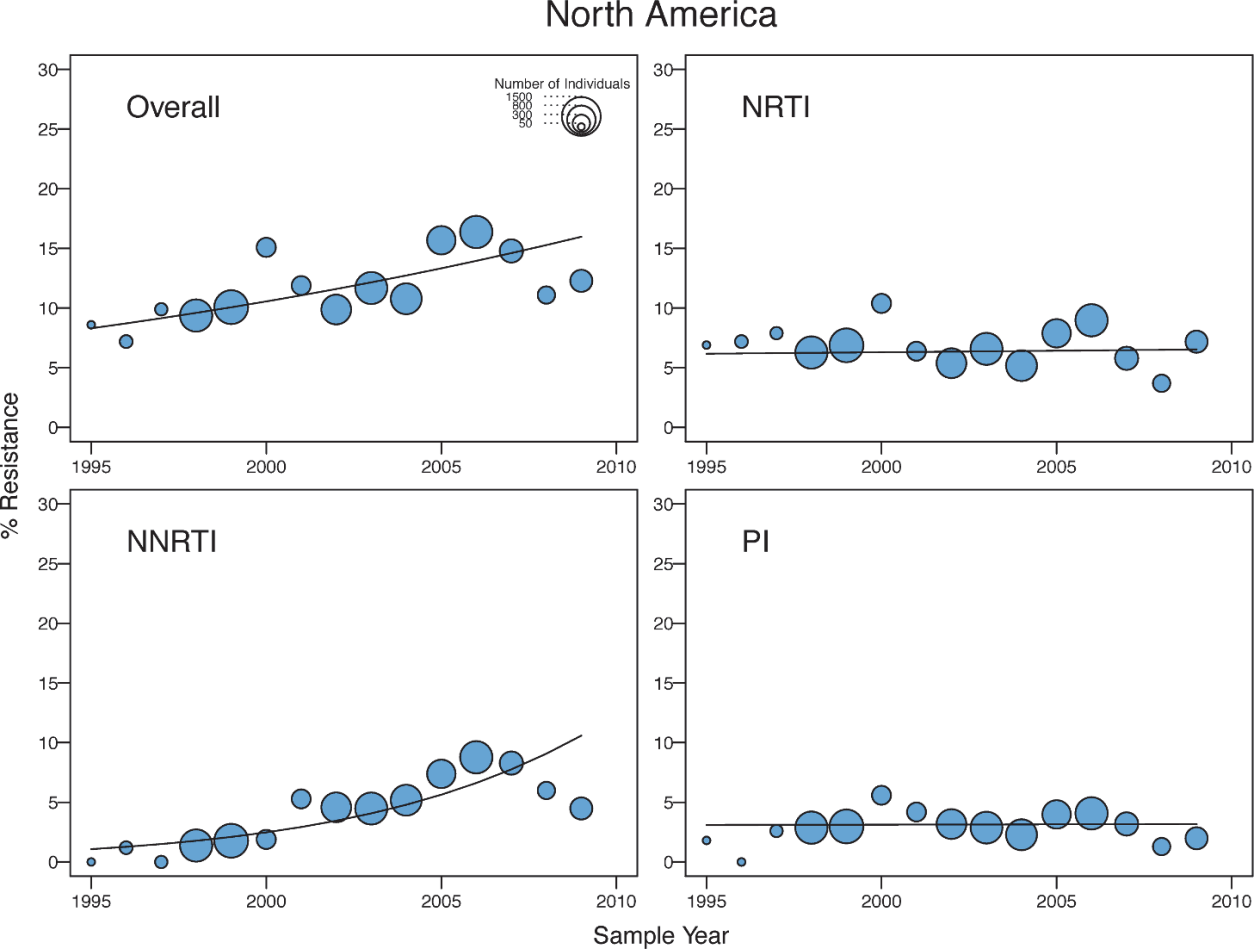
Temporal trends in the yearly proportion of individuals having one or more surveillance drug-resistance mutations in North America. The *x*-axes represent the calendar year of the sample. The diameter of each circle is proportional to the number of samples sequenced that year. The fitted line shows the fixed effect of sample year in generalized linear mixed model regression.

**Fig 7:**
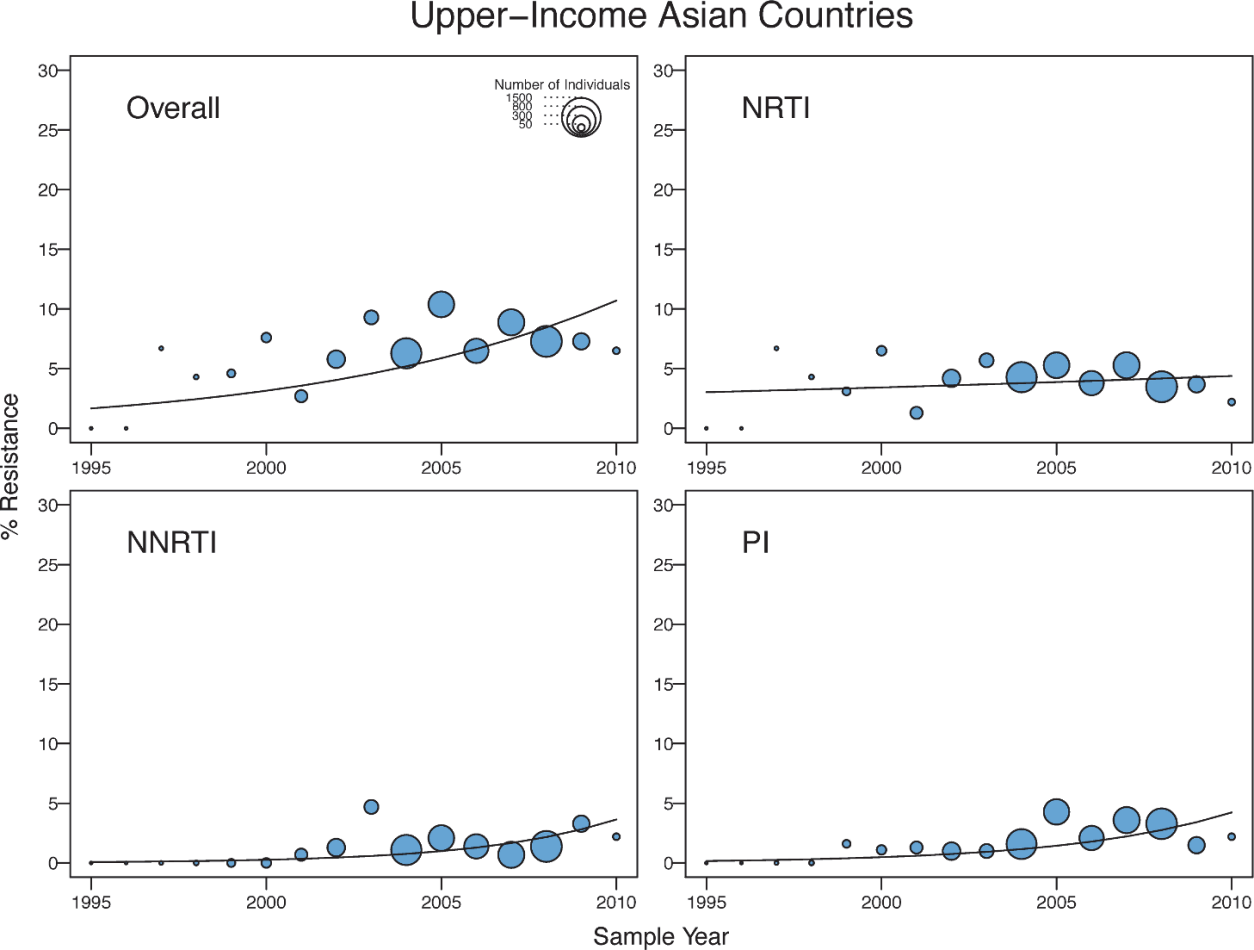
Temporal trends in the yearly proportion of individuals having one or more surveillance drug-resistance mutations in upper-income Asian countries. The *x*-axes represent the calendar year of the sample. The diameter of each circle is proportional to the number of samples sequenced that year. The fitted line shows the fixed effect of sample year in generalized linear mixed model regression.

There was a yearly 1.15-fold (95% CI: 1.07–1.23), 1.06-fold (95% CI: 1.00–1.11), and 1.05-fold (95% CI: 1.02–1.09) increase in the odds of overall TDR in the upper-income Asian countries, Latin America/Caribbean, and North America, respectively (Table 3). In Latin America/Caribbean and North America, the increase in overall TDR was accompanied by an increase in NNRTI-associated TDR (Figs 5 and 6). In the upper-income countries of Asia, the increase in the odds of overall TDR was accompanied by an increase in NNRTI- and PI-associated TDR (Fig 7). The temporal increase in the odds of PI-associated TDR in this region was partly attributable to two extended lineages in Japan published in two studies [25,26], one containing 30 individuals with viruses containing M46I alone and another containing 16 individuals with viruses containing M46L alone (S1 and S2 Figs).

**Fig 8:**
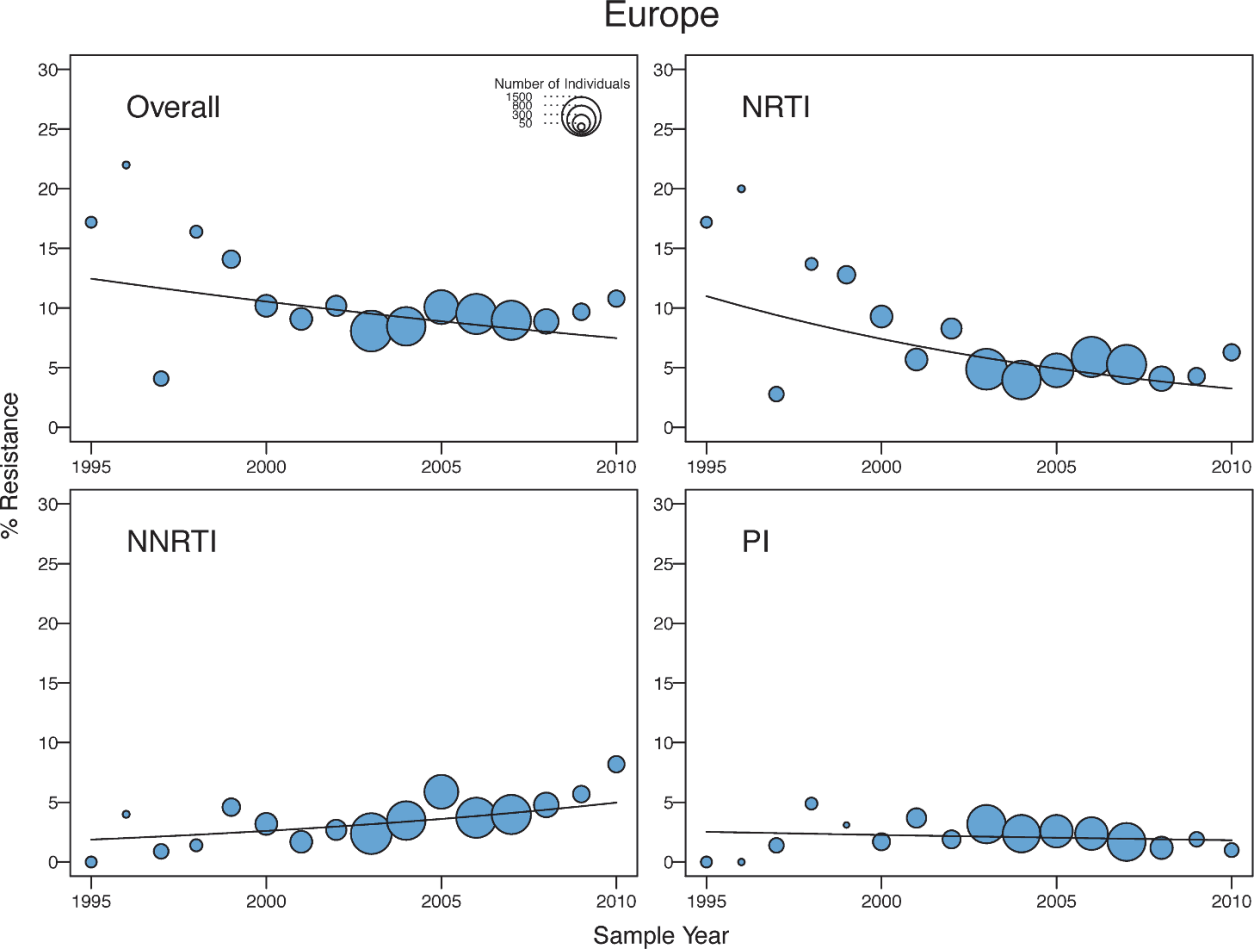
Temporal trends in the yearly proportion of individuals having one or more surveillance drug-resistance mutations in Europe (and Israel). The *x*-axes represent the calendar year of the sample. The diameter of each circle is proportional to the number of samples sequenced that year. The fitted line shows the fixed effect of sample year in generalized linear mixed model regression.

In Europe, there was a marginal yearly decrease in the odds of overall TDR (OR = 0.97; 95% CI: 0.93–1.00), accompanied by a yearly decrease in NRTI-associated TDR (OR = 0.93; 95% CI: 0.90–0.93) and a yearly increase in NNRTI-associated TDR (OR = 1.07; 95% CI: 1.01–1.13) (Fig 8). The decrease in overall TDR partly reflected the high levels of TDR in this region prior to 2000, in that a time trend analysis using only those virus samples obtained after 2000 did not show a significant change in the odds of overall TDR. In addition, the decrease resulted from a temporal increase in the proportion of viruses belonging to non-B subtypes, which were more likely to be from immigrants from LMICs. After adjusting for the presence of subtype B versus non-B subtypes, there was no yearly decrease in the odds of overall TDR, and the non-subtype-B viruses in Europe had significantly lower odds of TDR in any given year (OR = 0.5; 95% CI: 0.43–0.6; *p <* 0.001) than subtype B viruses.

With the exception of the distinction between subtype B versus non-subtype-B viruses in Europe, virus subtype in any region was not significantly associated with the odds of TDR, regardless of whether or not the model was adjusted for years since ARV scale-up or sampling year. An association between the duration of infection and the odds of TDR could not be adequately assessed because too few individuals had documented recent HIV-1 infection. In SSA, individuals recruited at a voluntary counseling and testing center (OR = 2.81; 95% CI: 1.92–4.12; *p* < 0.001) or HIV clinic (OR = 1.94; 95% CI: 1.47–2.55; *p* < 0.001) were more likely to have TDR than individuals recruited at other sites. In all other regions, there was no association between the odds of TDR and the recruitment site.

In SSA, SSEA, Latin America/Caribbean, Europe, North America, and the upper-income countries in Asia, 59% (6,766 of 11,536), 70% (4,576 of 6,522), 64% (3,614 of 5,628), 53% (6,312 of 11,802), 52% (4,853 of 9,283), and 72% (3,546 of 4,950) of virus sequences, respectively, contained mixtures at less than 0.5% of their nucleotide positions—a proxy for recent infection. A subset analysis using only virus sequences with less than 0.5% mixtures corroborated each of the trends in the main analysis (S2 Table).

A subset analysis using the 232 studies with sequentially recruited participants (80.8% of all 287 studies) corroborated each of the trends in the main analysis; the overall increase in TDR in Latin America/Caribbean and the overall decrease in TDR in Europe were not statistically significant in the subset analyses, even though the point estimates of the ORs were similar to those seen in the analysis including all studies (S3 Table).

### Correlation of Surveillance Drug-Resistance Mutations with Region

Of the 34 NRTI SDRMs, 16 occurred in ≥0.1% of the 50,870 viruses from all regions: most commonly M184V, the TAMs (M41L, D67G/N, K70R, L210W, T215F/Y, K219E/Q), the T215 revertants (T215C/D/E/S), T69D, and F77L. These 16 SDRMs comprised 234 (79%) of 298 NRTI SDRMs in SSA, 127 (69%) of 184 NRTI SDRMs in SSEA, 343 (90%) of 382 NRTI SDRMs in Latin America/Caribbean, and 2,462 (90%) of 2,724 NRTI SDRMs in the pooled upper-income countries. M184V and the TAMs were the most common NRTI SDRMs in all four regions (S4 Table). L74I (4.4%; eight of 184), V75M (8.2%; 15 of 184), and M184I (3.8%; seven of 184) accounted for a higher proportion of the NRTI SDRMs in SSEA than in other regions (<2% for each mutation in each of the other regions; *p* < 0.001). K70E (2.7%; eight of 298) accounted for a higher proportion of the NRTI SDRMs in SSA than in other regions (<0.3% in each of the other regions; *p* < 0.001). The T215 revertants accounted for a higher proportion of NRTI SDRMs in the pooled upper-income countries (25.2%; 685 of 2,724) and Latin America/Caribbean (18.9%; 72 of 382) than in SSA (4.7%; 14 of 298; *p* < 0.001) or SSEA (8.7%; 16 of 184; *p <* 0.001).

Of the 19 NNRTI SDRMs, four mutations—K101E, K103N, Y181C, and G190A—occurred in ≥0.1% of the 50,870 viruses from all regions. These four SDRMs comprised 80% or more of the NNRTI SDRMs in each of the four regions: 86% (264) of 306 NNRTI SDRMs in SSA, 81% (110) of 136 NNRTI SDRMs in SSEA, 81% (164) of 205 NNRTI SDRMs in Latin America/Caribbean, and 80% (910) of 1,140 NNRTI SDRMs in the pooled upper-income countries. K103N was the most common NNRTI SDRM in each region except for SSEA, accounting for 45% (137) of 306 NNRTI SDRMs in SSA, 49% (164) of 205 NNRTI SDRMs in Latin America/Caribbean, and 54% (612) of 1,140 NNRTI SDRMs in the pooled upper-income countries (S5 Table). Y181C was the most common NNRTI SDRM in SSEA, accounting for 32% (44) of 136 NNRTI SDRMs in this region.

Of the 40 PI SDRMs, nine were present in ≥0.1% of the 46,819 viruses from all regions. These nine SDRMs comprised 70% to 81% of PI SDRMs in each of the four regions: 72% (84) of 117 PI SDRMs in SSA, 81% (64) of 79 PI SDRMs in SSEA, 84% (131) of 156 PI SDRMs in Latin America/Caribbean, and 81% (795) of 986 PI SDRMs in the pooled upper-income countries. M46I/L, I85V, and L90M were the four most common PI SDRMs in SSA and SSEA, and among the six most common SDRMs in all regions. M46I was disproportionately more common in SSEA, where it accounted for 33% (26) of 79 PI SDRMs, compared with SSA (14%; 16 of 117; *p <* 0.001), Latin America/Caribbean (12%; 19 of 156; *p <* 0.001), and the pooled upper-income countries (16%, 159 of 795; *p <* 0.001). M46L was not associated with a region, accounting for 21% (24 of 117), 18% (14 of 79), 10% (16 of 156), and 12% (114 of 986) of PI SDRMs in SSA, SSEA, Latin America/Caribbean, and the pooled upper-income countries, respectively (S6 Table).

### Correlation of Surveillance Drug-Resistance Mutations with Subtype

Of the 34 NRTI SDRMs, the T215 revertants accounted for a higher proportion of NRTI SDRMs in subtype B viruses (24%; 725 of 2,920) than of viruses belonging to the remaining subtypes (9%; 57 of 634; *p <* 0.001) (S7 Table). V75M accounted for a higher proportion of NRTI SDRMs in CRF01_AE viruses compared with pooled viruses belonging to the remaining subtypes (10% versus 1%; 16 of 157 versus 25 of 3,397; *p <* 0.001).

Of the 19 NNRTI SDRMs, Y181C accounted for a higher proportion of NNRTI SDRMs in CRF01_AE viruses compared with pooled viruses belonging to the remaining subtypes (33% versus 13%; 38 of 115 versus 212 of 1,631; *p <* 0.001) (S8 Table). K103N accounted for a higher proportion of NNRTI SDRMs in subtype B viruses compared with pooled viruses belonging to the remaining subtypes (53% versus 40%; 646 of 1,212 versus 214 of 534; *p <* 0.001). P225H accounted for a higher proportion of NNRTI SDRMs in CRF02_AG viruses compared with pooled viruses belonging to the remaining subtypes (14% versus 3%; nine of 65 versus 48 of 1,681; *p <* 0.001). V106M accounted for a higher proportion of genotypic NNRTI SDRMs in subtype C viruses than in pooled viruses belonging to the remaining subtypes (5% versus 1%; eight of 179 versus ten of 1,567; *p <* 0.001).

Of the 40 PI SDRMs, L23I (16%; eight of 51) accounted for a higher proportion of PI SDRMs in subtype A viruses compared with pooled viruses belonging to the remaining subtypes (1%; 11 of 1,267; *p <* 0.001) (S9 Table). Of the eight subtype A viruses with L23I, six were part of a cluster of six sequences from one FSU study. F53Y accounted for a higher proportion of PI SDRMs in subtype CRF02_AG compared with pooled viruses belonging to the remaining subtypes (11% versus 1%; four of 37 versus seven of 1,281; *p <* 0.001). M46I, which was significantly more common in individuals from SSEA, was not significantly associated with any subtype. The 17 individuals with M46I in SSEA included 11 subtype CRF01_AE, four subtype C, and two subtype B viruses.

### Correlation of Surveillance Drug-Resistance Mutations with Their Prevalence in Treated Individuals

Among individuals with at least one SDRM, 21% of 501 individuals in SSA, 24% of 247 individuals in SSEA, 37% of 439 individuals in Latin America/Caribbean, and 36% of 2,508 individuals in the pooled upper-income countries had multiple SDRMs. In contrast, among ARV-experienced patients with at least one SDRM in HIVDB, 83% of 4,028 individuals in SSA, 92% of 1,880 individuals in SSEA, 92% of 3,458 individuals in Latin America/Caribbean, and 86% of 11,279 individuals in the pooled upper-income countries had more than one SDRM.

**Fig 9:**
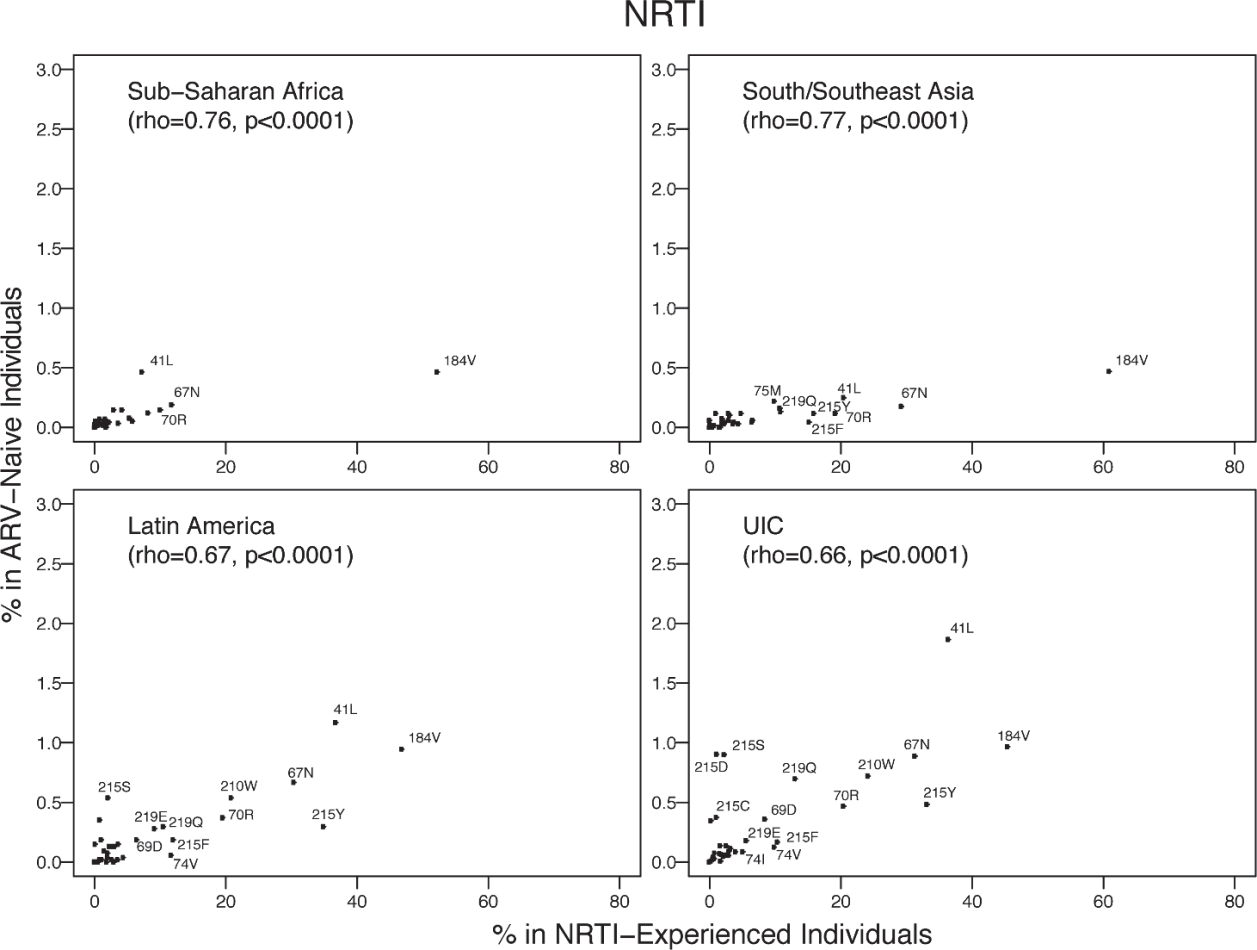
The prevalence of each NRTI-associated surveillance drug-resistance mutation in this meta-analysis versus in NRTI-experienced individuals in the same regions according to HIVDB. The Spearman’s rank correlation coefficient (rho) and the *p-*value are shown in each plot. The number of isolates from NRTI-experienced individuals were 4,522, 2,218, 4,164, and 13,522 for SSA, SSEA, Latin America/Caribbean, and the pooled upper-income countries (UIC; Europe, North America, and upper-income Asian countries), respectively.

The proportion of individuals with each NRTI SDRM was highly correlated with published proportions of these mutations in NRTI-experienced individuals from the same region: SSA, rho = 0.76 (*p <* 0.001); SSEA, rho = 0.77 (*p <* 0.001); Latin America/Caribbean, rho = 0.67 (*p <* 0.001); and the pooled upper-income countries, rho = 0.66 (*p <* 0.001) (Fig 9). The mean proportions of the five most common NRTI SDRMs in NRTI-treated individuals was 65-fold higher than their proportions in ARV-naïve individuals in SSA, 136-fold higher than in ARV-naïve individuals in SSEA, 41-fold higher than in ARV-naïve individuals in Latin America/Caribbean, and 57-fold higher than in ARV-naïve individuals in the pooled upper-income countries.

**Fig 10:**
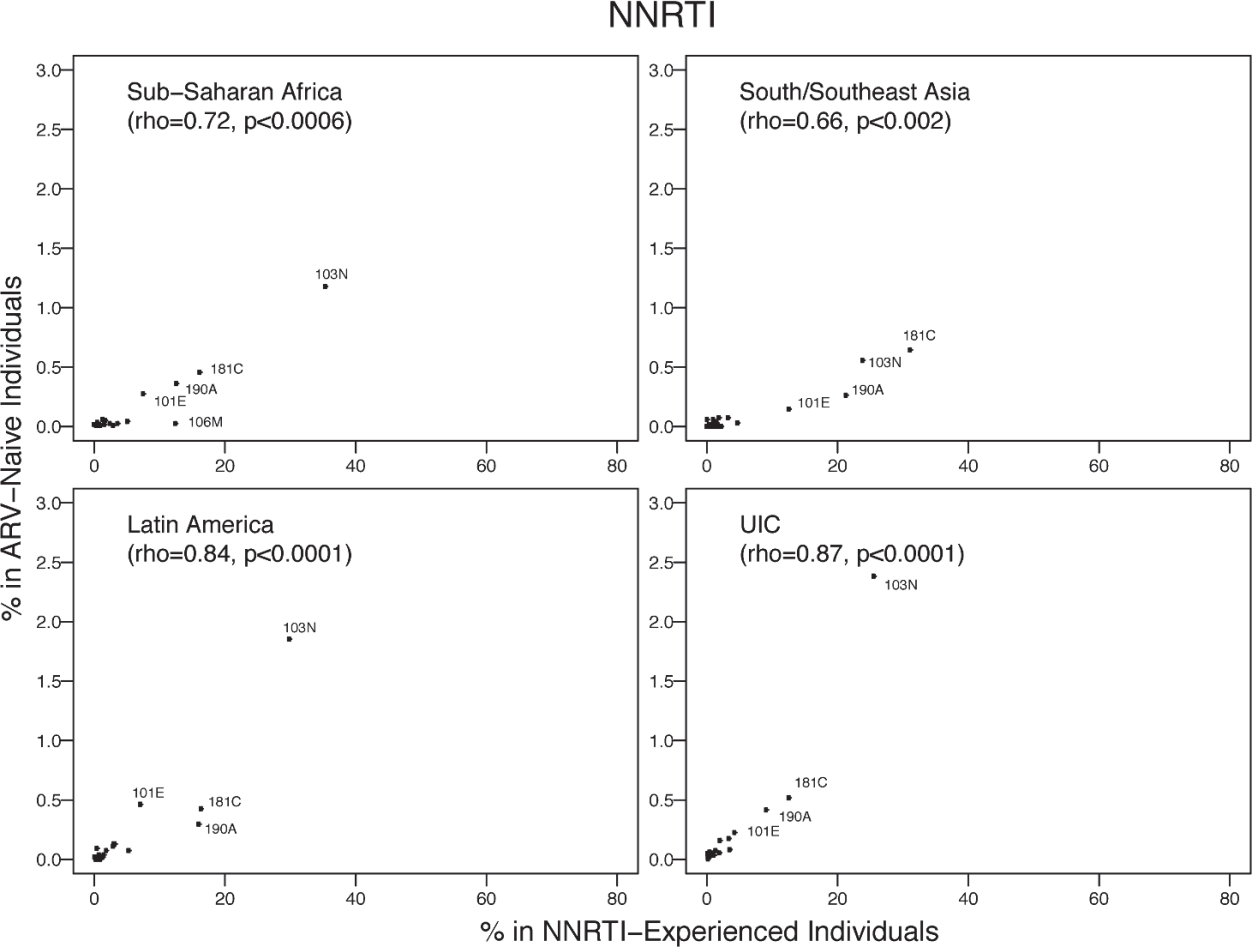
The prevalence of each NNRTI-associated surveillance drug-resistance mutation in this meta-analysis versus in NNRTI-experienced individuals in the same regions according to HIVDB. The Spearman’s rank correlation coefficient (rho) and the *p-*value are shown in each plot. The number of isolates from NNRTI-experienced individuals were 4,959, 1,994, 3,677, and 8,927 for SSA, SSEA, Latin America/Caribbean, and the pooled upper-income countries (UIC; Europe, North America, and upper-income Asian countries), respectively.

The proportion of individuals with each NNRTI SDRM was highly correlated with published proportions of these mutations in NNRTI-experienced individuals from the same region: SSA, rho = 0.72 (*p <* 0.001); SSEA, rho = 0.66 (*p* = 0.002); Latin America/Caribbean, rho = 0.84 (*p <* 0.001); and the pooled upper-income countries, rho = 0.87 (*p <* 0.001) (Fig 10). The mean proportion of the five most common NNRTI SDRMs in NNRTI-treated individuals was 85-fold higher than their proportion in ARV-naïve individuals in SSA, 122-fold higher than in ARV-naïve individuals in SSEA, 24-fold higher than in ARV-naïve individuals in Latin America/Caribbean, and 39-fold lower in ARV-naïve individuals in the pooled upper-income countries.

**Fig 11:**
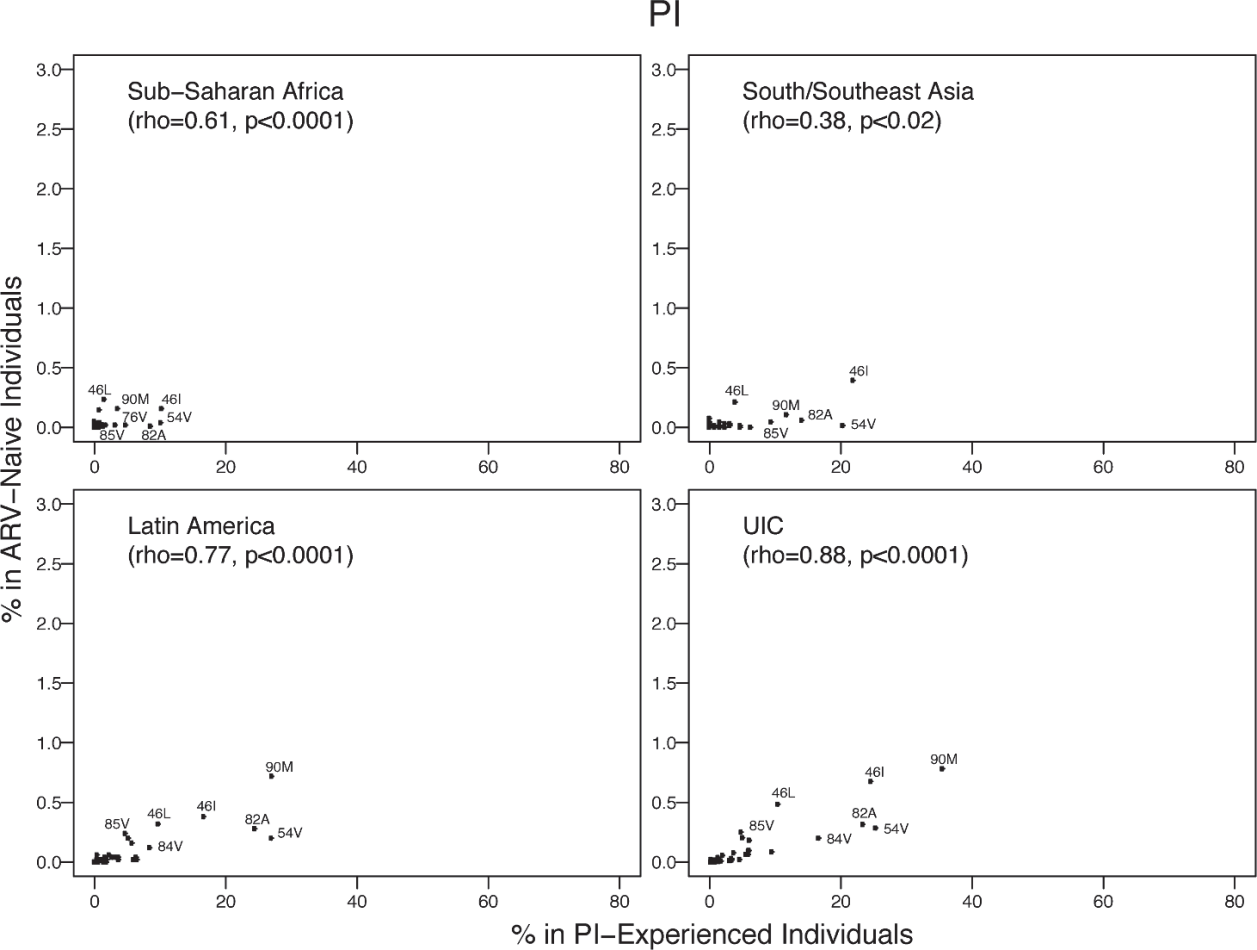
The prevalence of each PI-associated surveillance drug-resistance mutation in this meta-analysis versus in PI-experienced individuals in the same regions according to HIVDB. The Spearman’s rank correlation coefficient (rho) and the *p-*value are shown in each plot. The number of isolates from PI-experienced individuals were 717, 103, 4,107, and 9,985 for SSA, SSEA, Latin America/Caribbean, and the pooled upper-income countries (UIC; Europe, North America, and upper-income Asian countries), respectively.

The proportion of individuals with each PI SDRM was correlated with published proportions of these mutations in PI-treated individuals from the same region: SSA, rho = 0.61 (*p <* 0.001); SSEA, rho = 0.38 (*p* = 0.02); Latin America/Caribbean, rho = 0.77 (*p <* 0.001), and the pooled upper-income countries, rho = 0.88 (*p <* 0.001) (Fig 11). The mean proportion of the five most common PI SDRMs in PI-treated individuals was 291-fold higher than their proportion in ARV-naïve individuals in SSA, 388-fold higher than in ARV-naïve individuals in SSEA, 66-fold higher than in ARV-naïve individuals in Latin America/Caribbean, and 65-fold higher than in ARV-naïve individuals in the pooled upper-income countries. In all regions, the proportion of PI-treated individuals with M46L or I85V divided by the number of ARV-naïve individuals with these SDRMs was much lower than the same proportion for all other commonly occurring SDRMs.

### Correlation of Surveillance Drug-Resistance Mutations with Estimated Levels of Genotypic Resistance

**Fig 12:**
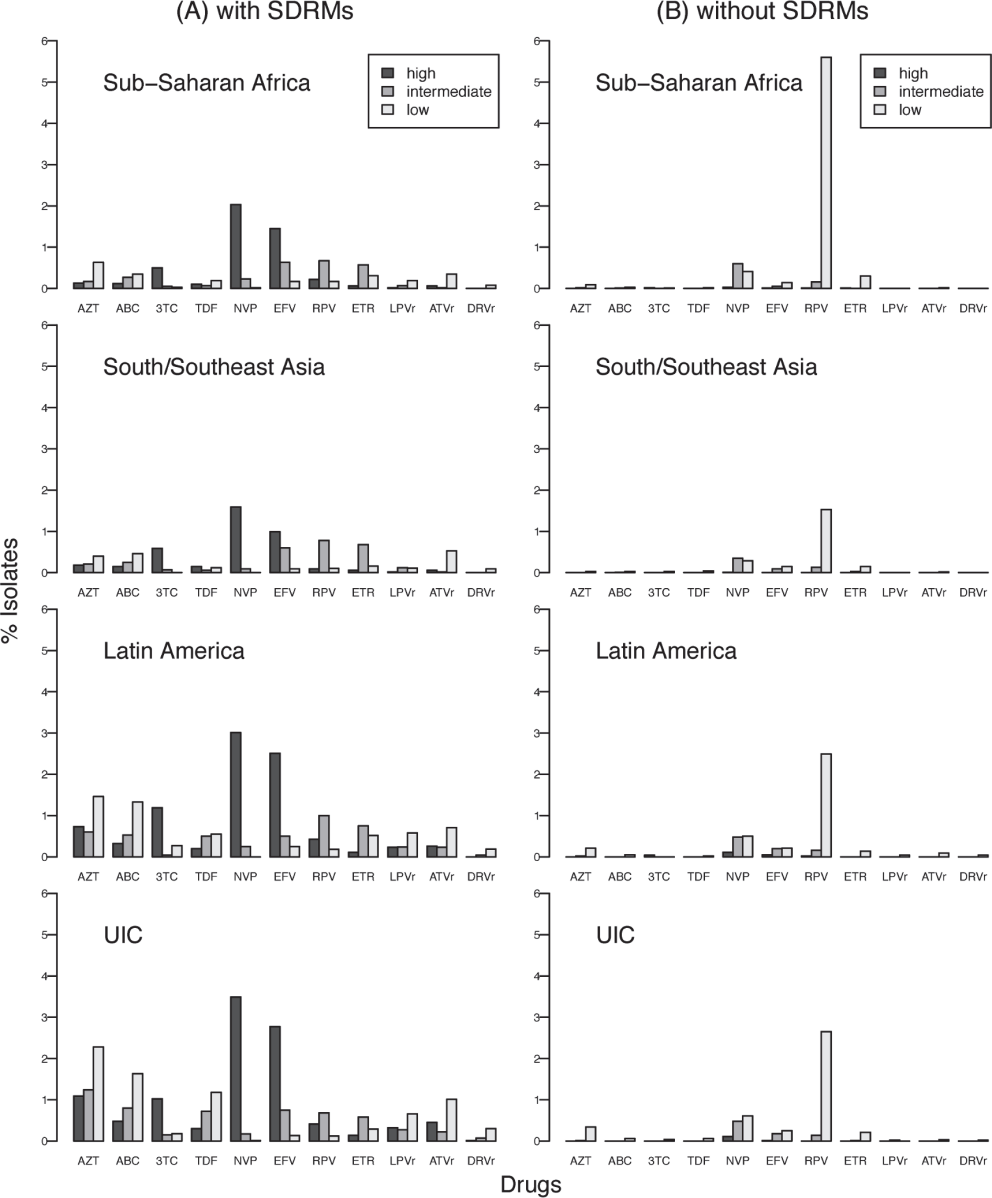
Estimated levels of predicted genotypic drug resistance for viruses with and without surveillance drug-resistance mutations. HIVDB genotypic resistance interpretation program predictions of NRTI, NNRTI, and PI resistance for all virus samples using NRTI, NNRTI, and PI SDRMs, respectively (A). HIVDB program predictions of NRTI, NNRTI, and PI resistance in all samples without an SDRM (B). NRTIs: zidovudine (AZT), abacavir (ABC), lamivudine (3TC), and tenofovir (TDF); NNRTIs: nevirapine (NVP), efavirenz (EFV), rilpivirine (RPV), and etravirine (ETR); PIs: lopinavir (LPVr), atazanavir (ATVr), and darunavir (DRVr). UIC, upper-income countries.

Fig 12A shows the prevalence of resistance predicted by the HIVDB genotypic resistance interpretation program to the NRTIs zidovudine, abacavir, lamivudine, and tenofovir using the NRTI SDRMs; to the NNRTIs nevirapine, efavirenz, rilpivirine, and etravirine using the NNRTI SDRMs; and to the PIs lopinavir, atazanavir, and darunavir using the PI SDRMs.

Predicted NRTI resistance ranged from 0.4% (tenofovir; 41 of 11,536) to 0.9% (zidovudine; 108 of 11,536) in SSA, 0.3% (tenofovir; 22 of 6,522) to 0.8% (zidovudine; 53 of 6,522) in SSEA, and 1.4% (lamivudine; 419 of 30,663) to 4.2% (zidovudine; 1,297 of 30,663) in the pooled upper-income countries and Latin America/Caribbean. Predicted lamivudine resistance was usually high-level, caused by M184V/I. Predicted resistance to the other NRTIs was usually low or intermediate. Predicted NNRTI resistance ranged from 0.9% (etravirine; 109 of 11,536) to 2.3% (nevirapine; 261 of 11,536) in SSA, 0.9% (etravirine; 61 of 6,522) to 1.7% (nevirapine and efavirenz; 114 of 6,522) in SSEA, and 1.1% (etravirine; 326 of 30,663) to 3.6% (nevirapine; 1,089 of 30,663) in the pooled upper-income countries and Latin America/Caribbean. Nearly all nevirapine resistance and about two-thirds of efavirenz resistance was predicted to be high-level. Etravirine and rilpivirine resistance was usually caused by Y181C, a mutation selected primarily by nevirapine. Predicted lopinavir, atazanavir, and darunavir resistance was less than 0.5% in SSA and SSEA. In Latin America/Caribbean and the pooled upper-income countries, the estimated prevalence of lopinavir, atazanavir, and darunavir resistance was nearly 1%.

Fig 12B shows that few of the 48,722 virus samples without an NRTI SDRM were predicted to have reduced NRTI susceptibility. However, among the 48,722 samples without NNRTI SDRMs, 5.6% (644 of 11,536) of samples in SSA, 1.6% (104 of 6,522) in SSEA, and 2.6% (655 of 25,035) in the pooled upper-income countries were predicted to have low-level rilpivirine resistance as a result of the polymorphic mutation E138A, which occurs in up to 6% of subtype A and C viruses [34,35]. Nevirapine and efavirenz resistance were predicted in about 1% and 0.5% of virus samples without NNRTI SDRMs as a result of several minimally polymorphic (e.g., A98G, V108I, and V179D) and rare nonpolymorphic (e.g., E138K, G190Q, F227C, and K238T) NNRTI-resistance mutations. Many of the 45,883 samples without PI SDRMs had accessory polymorphic PI-resistance mutations. However, few samples had sufficient numbers of these accessory mutations to reduce lopinavir, atazanavir, or darunavir susceptibility.

### Molecular Phylogenetics

In studies conducted in SSA, Latin America/Caribbean, North America, Europe, and SSEA, the median sequence dissimilarity index was 98%, 96%, 95%, 84%, and 80%, respectively (S3 Fig). In the upper-income countries of Asia, the median sequence dissimilarity index was 65%. In FSU countries, the median sequence dissimilarity index was 35%. Overall, 67 studies had two or more closely related sequences with identical SDRMs. There would have been a median 1.1% lower TDR prevalence in these studies had only one sequence from each set of closely related sequences been included in the analysis.

No study in SSA or SSEA contained an SDRM cluster (defined in the Methods as a set of three or more closely related sequences with identical SDRMs), and only 19 pairs (5%; 38 of 763 viruses with TDR) of closely related sequences contained an identical SDRM in these two regions. In Latin America/Caribbean, one study contained an SDRM cluster of three viruses with the NNRTI SDRM K103N. In FSU countries, there was one SDRM cluster of six viruses with the PI SDRM L23I, a nelfinavir-resistance mutation. In North America, Europe, and the upper-income countries of Asia there were 22, 21, and 19 SDRM clusters, respectively. In these three regions, the NNRTI SDRM K103N alone occurred in 22 clusters (96 individuals), a NRTI SDRM T215 revertant alone occurred in 16 clusters (82 individuals), and the NNRTI SDRM G190A alone occurred in five clusters (17 individuals). In addition to the large Japanese cluster of PI SDRM M46I (30 individuals), there was one SDRM cluster of viruses from five individuals in North America with this mutation. There were seven SDRM clusters involving 39 individuals with more than one SDRM.

## Discussion

This is to our knowledge the first individual-patient-level meta-analysis of TDR in HIV-1-infected, ARV-naïve populations. HIV-1 RT (with or without protease) sequences from more than 50,000 individuals from 287 studies were analyzed for geo-temporal trends in TDR prevalence using identical analytical methods for quality control, molecular phylogenetics, mutational patterns, and predicted clinical significance. The availability of sequences from each study participant made it possible to characterize the patterns of drug-resistance mutations in individuals from different regions and in viruses of different subtypes, and to analyze how often the same drug-resistance mutations were present in closely related virus sequences.

The 287 studies in this meta-analysis included 125 published studies of 25 or more ARV-naïve individuals included in two previous meta-analyses [13,14] and 162 additional studies, including 85 published between 2011 and 2013. HIV-1 RT sequence data were not available for 117 studies included in the two previous meta-analyses, including 22 studies from SSA, nine from SSEA, ten from Latin America/Caribbean, 50 from Europe, 24 from North America, two from upper-income Asian countries, and one from Australia. Therefore, of the combined 404 studies in this and the two previous meta-analyses, this meta-analysis includes 81% (95/117), 86% (56/65), 79% (38/48), 46% (42/92), 53% (27/51), and 86% (12/14) of the studies from SSA, SSEA, Latin America/Caribbean, Europe, North America, and the upper-income Asian countries, respectively. In 2012, WHO published a report summarizing the results of 82 surveys of 3,588 individuals in 30 LMICs between 2004 and 2010 using the WHO HIV Drug Resistance Threshold Survey targeting individuals recently infected with HIV-1 [11,36]. Sequences for 21 of these surveys were publicly available by February 2014 and were included in this meta-analysis.

The median overall TDR prevalence in SSA and SSEA was 2.8% and 2.9%, respectively. There was an estimated 1.1-fold yearly increase in the odds of overall, NRTI-associated, and NNRTI-associated TDR in SSA since ARV scale-up began. In contrast, there was no significant temporal change in the odds of TDR in SSEA since ARV scale-up began. The median overall TDR prevalence in upper-income Asian countries, Latin America/Caribbean, Europe, and North America was 5.6%, 7.6%, 9.4%, and 11.5%, respectively. In both North America and Latin America/Caribbean, there was an estimated 1.1-fold yearly increase in the odds of overall TDR and a 1.2-fold yearly increase in the odds of NNRTI-associated TDR. In the upper-income Asian countries, there was an estimated 1.2-fold yearly increase in the odds of overall TDR and a 1.3-fold yearly increase in the odds of NNRTI- and PI-associated TDR. In Europe, there was a 0.9-fold yearly decrease in the odds of NRTI-associated TDR and a 1.1-fold yearly increase in the odds of NNRTI-associated TDR.

A major limitation of the studies in our meta-analysis is their heterogeneity with respect to the duration of infection prior to virus sampling. Many drug-resistance mutations reduce HIV-1 replication fitness and recede to levels not detectable by standard genotypic resistance testing in the absence of selective drug pressure. This occurs rapidly for the NRTI-resistance mutation M184V, which recedes to undetectable levels at a rate of about 50% per year [37,38]. It occurs at a much slower rate of about 10% to 20% per year for most NNRTI-resistance mutations and most TAMs [37,38]. Indeed, in our analysis, there was a particularly high correlation between the prevalence of NNRTI SDRMs in ARV-naïve and ARV-experienced individuals in the same region likely reflect the increased fitness, and hence stability, of NNRTI-resistance mutations [39].

Several studies have shown that the proportion of sequence positions with a nucleotide mixture increases with the duration of infection [26–28]. In our study, we found that more than one-half of the sequences from studies of recently infected individuals did not contain a nucleotide mixture. The highest levels of nucleotide mixtures were in studies of individuals presenting to an HIV clinic whereas intermediate levels of nucleotide mixtures were detected among blood donors, antenatal clinic attendees, and VCT attendees. An analysis limited to only those sequences with less than 0.5% mixed nucleotides—a proxy for recent infection—yielded comparable trends to those obtained using the complete dataset reinforcing the trends reported in this meta-analysis.

Endemic TDR strains emanating from a single instance of ARV-selection pressure that spread among many individuals have different public health implications from TDR strains emanating from multiple independent episodes of ARV-selection pressure [40–42]. Endemic strains may carry a greater risk of ongoing transmission reflecting their ability to persist in a population in the absence of selective drug pressure. In contrast, increasing TDR resulting from multiple separate episodes of ARV-selection pressure can be mitigated by reducing the risk of virological failure in patients on therapy.

To study whether TDR strains were likely to have arisen independently, we estimated the extent of sequence clustering in each study and determined whether each drug-resistant virus was part of a sequence cluster that contained other viruses with the same mutation. Of the 763 drug-resistant variants in SSA and SSEA, 19 pairs of viruses (*n =* 38; 5%) were closely related to one another. In contrast, the remaining 725 viruses (95%) were not closely related to one another. Although many of these viruses may be closely related to viruses that were not sampled, phylogenetic analysis of the sequences in each study from SSA and SSEA suggests that most TDR variants in this meta-analysis arose independently.

In SSA and SSEA, 89% of NNRTI-associated SDRMs were associated with high-level resistance to nevirapine or efavirenz, whereas only 27% of NRTI SDRMs was associated with high-level resistance to zidovudine, lamivudine, tenofovir, or abacavir. Several studies also suggest that transmitted NNRTI resistance is more likely than transmitted NRTI resistance to cause virological failure on a first-line NRTI/NNRTI-containing regimen [15,16,20,43,44]. Should NNRTI-associated TDR continue to increase, the inability to predict whether patients will respond to an initial NRTI/NNRTI-containing regimen would undermine confidence in the treatability of HIV-1 in LMICs and weaken the HIV care continuum. The point at which such a loss of confidence would occur is difficult to predict but it would likely occur well below thresholds at which cost-effectiveness models predict that a reduction in efficacy for entire populations [45].

The resources and capacity to perform HIV-1 drug resistance testing in LMICs are limited and, where available, are concentrated in a few central laboratories. In addition, the infrastructure in many LMICs does not support the expansion in the number of these laboratories or the rapid transportation of samples to these laboratories. The finding that a few mutations were responsible for 80% of NNRTI-associated TDR in all regions and subtypes should motivate the development of inexpensive point-of-care point mutation assays for use in LMIC regions [46,47]. Even in the context of a public health approach to ARV therapy, where few standardized regimens are available at the population level, a reliable point-of-care genotypic resistance test could identify which patients should receive standard first-line therapy and which should receive a PI-containing regimen.

TDR surveillance of both newly infected individuals and patients presenting for ARV therapy informs treatment guidelines and diagnostic strategies particularly in regions where routine genotypic resistance testing is not affordable [48]. This study demonstrates that sequence analysis is an important component of TDR surveillance because it yields insights into the molecular epidemiology of TDR and the specific drug-resistance mutations responsible for TDR. The finding that most of the TDR strains in SSA and SSEA arose independently suggests that the use of ARV regimens with a high genetic barrier to resistance combined with improved patient adherence will mitigate the increase in TDR by reducing the generation of new ARV-resistant strains [49]. The finding that a few NNRTI-resistance mutations were responsible for most cases of transmitted high-level resistance suggests that inexpensive diagnostic point-mutation assays for these NNRTI-resistance mutations may be useful for pre-therapy screening in those LMIC regions with the highest levels of TDR.

## Supporting Information

S1 Fig Time-scaled analysis indicating an extended lineage of 30 individuals with viruses containing the PI SDRM M46I from two studies conducted in Japan. Bayesian phylogenetic inference of time-measured trees was performed using Markov chain Monte Carlo (MCMC) sampling implemented in BEAST v.1.8.0. The substitution process was modeled according to the general time-reversible substitution model with discrete gamma rate variation among sites. An exponential growth model was specified as coalescent tree prior. Independent MCMC analyses were run for 10 million generations, sampling every 5,000 generations, and the first 10% of the samples was discarded as burn-in before combining the samples. The runs were investigated based on effective sample size calculated using Tracer. Then a maximum clade credibility tree was selected from the posterior tree distribution and visualized using FigTree. The values at the nodes represent posterior support values (posterior probability) for the clusters. Viruses containing M46I are labeled in red.
(EPS)

S2 Fig Time-scaled analysis indicating an extended lineage of 16 individuals with viruses containing the PI SDRM M46L alone from two studies conducted in Japan. Bayesian phylogenetic inference of time-measured trees was performed using MCMC sampling implemented in BEAST v.1.8.0. The substitution process was modeled according to the general time-reversible substitution model with discrete gamma rate variation among sites. An exponential growth model was specified as coalescent tree prior. Independent MCMC analyses were run for 10 million generations, sampling every 5,000 generations, and the first 10% of the samples was discarded as burn-in before combining the samples. The runs were investigated based on effective sample size calculated using Tracer. Then a maximum clade credibility tree was selected from the posterior tree distribution and visualized using FigTree. The values at the nodes represent posterior support values (posterior probability) for the clusters. Viruses containing M46L are labeled in red.
(EPS)

S3 Fig The sequence dissimilarity index for each study by region. The sequence dissimilarity index was defined in the Methods. The horizontal line indicates the median sequence dissimilarity index of the studies in each region. Regions are SSA, SSEA, Latin America/Caribbean, FSU, upper-income Asian countries (UIC-Asia), Europe, and North America.
(EPS)

S1 Table Summary of each of the 287 studies. Data provided include a reference, number of study participants, countries, recruitment method, median sample year, study purpose, recruitment site, virus subtype distribution, overall TDR (percent), NRTI TDR (percent), NNRTI TDR (percent), a link to CPR analysis output, and the number of individuals having each SDRM.
(XLSX)

S2 Table Yearly change in odds of TDR in generalized linear mixed regression models in regions with and without ARV scale-up using samples containing <0.5% mixed positions. (DOCX)

S3 Table Yearly change in odds of TDR in generalized linear mixed regression models in regions with and without ARV scale-up using samples from studies of sequentially recruited individuals. (DOCX)

S4 Table Proportion of each NRTI SDRM by region. (DOCX)

S5 Table Proportion of each NNRTI SDRM by region. (DOCX)

S6 Table Proportion of each PI SDRM by region. (DOCX)

S7 Table Proportion of each NRTI SDRM by virus subtype. (DOCX)

S8 Table Proportion of each NNRTI SDRM by virus subtype. (DOCX)

S9 Table Proportion of each PI SDRM by virus subtype. (DOCX)

S1 Text PRISMA checklist. (DOC)

## Abbreviations

ARV: antiretroviral
CPR: Calibrated Population Resistance
FSU: former Soviet Union
HIVDB: HIV Drug Resistance Database
IQR: interquartile range
LMIC: low- and middle-income country
MCMC: Markov chain Monte Carlo
NNRTI: non-nucleoside reverse transcriptase inhibitor
NRTI: nucleoside reverse transcriptase inhibitor
OR: odds ratio
PI: protease inhibitor
RT: reverse transcriptase
SDRM: surveillance drug-resistance mutation
SSA: sub-Saharan Africa
SSEA: south/southeast Asia
TAM: thymidine-analog mutation
TDR: transmitted drug resistance
