# Correction: Geographic and Temporal Trends in the Molecular Epidemiology and Genetic Mechanisms of Transmitted HIV-1 Drug Resistance: An Individual-Patient- and Sequence-Level Meta-Analysis

**DOI:** 10.1371/journal.pmed.1001845

**Published:** 2015-06-01

**Authors:** Soo-Yon Rhee, Jose Luis Blanco, Michael R. Jordan, Jonathan Taylor, Philippe Lemey, Vici Varghese, Raph L. Hamers, Silvia Bertagnolio, Tobias F. Rinke de Wit, Avelin F. Aghokeng, Jan Albert, Radko Avi, Santiago Avila-Rios, Pascal O. Bessong, James I. Brooks, Charles A. B. Boucher, Zabrina L. Brumme, Michael P. Busch, Hermann Bussmann, Marie-Laure Chaix, Bum Sik Chin, Toni T. D’Aquin, Cillian F. De Gascun, Anne Derache, Diane Descamps, Alaka K. Deshpande, Cyrille F. Djoko, Susan H. Eshleman, Herve Fleury, Pierre Frange, Seiichiro Fujisaki, P. Richard Harrigan, Junko Hattori, Africa Holguin, Gillian M. Hunt, Hiroshi Ichimura, Pontiano Kaleebu, David Katzenstein, Sasisopin Kiertiburanakul, Jerome H. Kim, Sung Soon Kim, Yanpeng Li, Irja Lutsar, Lynn Morris, Nicaise Ndembi, Peng NG Kee, Ramesh S. Paranjape, Martine Peeters, Mario Poljak, Matt A. Price, Manon L. Ragonnet-Cronin, Gustavo Reyes-Terán, Morgane Rolland, Sunee Sirivichayakul, Davey M. Smith, Marcelo A. Soares, Vincent V. Soriano, Deogratius Ssemwanga, Maja Stanojevic, Mariane A. Stefani, Wataru Sugiura, Somnuek Sungkanuparph, Amilcar Tanuri, Kok Keng Tee, Hong-Ha M. Truong, David A. M. C. van de Vijver, Nicole Vidal, Chunfu Yang, Rongge Yang, Gonzalo Yebra, John P. A. Ioannidis, Anne-Mieke Vandamme, Robert W. Shafer

The first author is missing an affiliation. She is also affiliated with #5 KU Leuven—University of Leuven, Department of Microbiology and Immunology, Rega Institute for Medical Research, Clinical and Epidemiological Virology, Leuven, Belgium.

The following information is missing from the funding statement: AMV is supported by Fonds voor Wetenschappelijk Onderzoek—Flanders (FWO) grant G.0692.14

## References

[1] Rhee S-Y, Blanco JL, Jordan MR, Taylor J, Lemey P, Varghese V, et al (2015) Geographic and Temporal Trends in the Molecular Epidemiology and Genetic Mechanisms of Transmitted HIV-1 Drug Resistance: An Individual-Patient- and Sequence-Level Meta-Analysis. PLoS Med 12(4): e1001810 doi:10.1371/journal.pmed.1001810 25849352 10.1371/journal.pmed.1001810 PMC4388826

