# Supplementary material for: Geographic and Temporal Trends in the Molecular Epidemiology and Genetic Mechanisms of Transmitted HIV-1 Drug Resistance: An Individual-Patient- and Sequence-Level Meta-Analysis
Source: PLoS Med. 2015 Apr 7;12(4):e1001810. doi: 10.1371/journal.pmed.1001810 (PMC4388826; doi:10.1371/journal.pmed.1001810)
Supplement: S2 Table — (DOCX) [file pmed.1001810.s005.docx]

| S2 Table. Yearly Change in Odds of Transmitted Drug Resistance (TDR) in Generalized Linear Mixed Regression Models in Regions with and without ARV Scale-up*^a^* using Samples Containing <0.5% Mixed Positions | | | |
| --- | --- | --- | --- |
| Region | Drug class | OR*^b^*  (95% CI) | p-value*^b^* |
| *OR for years since ARV scale-up^c^* | | | |
| Sub-Saharan Africa  (n=6,766) | Overall | 1.11 (1.10-1.11) | <0.001 |
|  | NRTI | 1.11 (1.03-1.20) | 0.007 |
|  | NNRTI | 1.14 (1.08-1.21) | <0.001 |
|  | PI | 1.03 (0.96-1.10) | 0.5 |
| South/Southeast Asia  (n=4,576) | Overall | 0.98 (0.92-1.05) | 0.5 |
|  | NRTI | 0.95 (0.85-1.05) | 0.3 |
|  | NNRTI | 1.09 (0.96-1.23) | 0.2 |
|  | PI | 0.98 (0.89-1.08) | 0.6 |
| *OR for sample year^c^* | | | |
| Latin America  (n=3,614) | Overall | 1.05 (0.99-1.11) | 0.07 |
|  | NRTI | 0.99 (0.92-1.06) | 0.7 |
|  | NNRTI | 1.17 (1.17-1.18) | <0.001 |
|  | PI | 1.01 (0.94-1.09) | 0.8 |
| Europe  (n=6,312) | Overall | 0.96 (0.93-1.00) | 0.05 |
|  | NRTI | 0.92 (0.88-0.96) | <0.001 |
|  | NNRTI | 1.07 (1.00-1.13) | 0.04 |
|  | PI | 0.99 (0.93-1.08) | 0.99 |
| North America  (n=4,853) | Overall | 1.05 (1.01-1.10) | 0.01 |
|  | NRTI | 0.98 (0.93-1.04) | 0.6 |
|  | NNRTI | 1.19 (1.12-1.28) | <0.001 |
|  | PI | 0.98 (0.93-1.04) | 0.6 |
| Upper-Income Asian Countries  (n=3,546) | Overall | 1.13 (1.03-1.23) | 0.007 |
|  | NRTI | 0.97 (0.91-1.04) | 0.4 |
|  | NNRTI | 1.40 (1.15-1.70) | <0.001 |
|  | PI | 1.22 (1.05-1.41) | 0.01 |
| ^a^Three studies from North Africa and two studies from Australia excluded. The region “Latin America” includes three studies from Caribbean countries.  ^b^For each region, a generalized linear mixed model was used to assess the yearly change in the odds (OR) of TDR accounting for study heterogeneity using the R package lme4. The model included the categorical outcome variable indicating the presence or absence of TDR and the two explanatory variables years since scale-up (or the sample year) as a fixed-effect term and the study as a random-effect term.  ^c^Yearly change in the odds of TDR since ARV scale-up in regions with national ARV scale-up programs and each sample year in regions without national ARV scale-up; the number of individuals is indicated in each region (n). | | | |
