# Supplementary material for: Geographic and Temporal Trends in the Molecular Epidemiology and Genetic Mechanisms of Transmitted HIV-1 Drug Resistance: An Individual-Patient- and Sequence-Level Meta-Analysis
Source: PLoS Med. 2015 Apr 7;12(4):e1001810. doi: 10.1371/journal.pmed.1001810 (PMC4388826; doi:10.1371/journal.pmed.1001810)
Supplement: S4 Table — (DOCX) [file pmed.1001810.s007.docx]

| S4 Table. Proportion of each NRTI SDRM According to Region*^a^* | | | | | |
| --- | --- | --- | --- | --- | --- |
| SDRM | Sub-Saharan Africa  (n=298)  % | South/ Southeast Asia  (n=184)  % | Latin America  (n=382)  % | Upper-Income Country Regions  (n=2,724)  % | All  Regions  (n=3,588)  % |
| M41L | 18 (54) | 9 (17) | 16 (63) | 18 (479) | 17 (613) |
| M184V | 18 (54) | 17 (32) | 13 (51) | 9.1 (248) | 11 (385) |
| D67N | 7.4 (22) | 6.5 (12) | 9.4 (36) | 8.4 (228) | 8.3 (298) |
| T215S | 2 (6) | 4.4 (8) | 7.6 (29) | 8.5 (231) | 7.6 (274) |
| T215D | 1 (3) | 1.1 (2) | 5 (19) | 8.5 (232) | 7.1 (256) |
| L210W | 5.7 (17) | 4.9 (9) | 7.6 (29) | 6.8 (185) | 6.7 (240) |
| K219Q | 5.7 (17) | 6 (11) | 4.2 (16) | 6.6 (179) | 6.2 (223) |
| K70R | 5.7 (17) | 4.4 (8) | 5.2 (20) | 4.4 (120) | 4.6 (165) |
| T215Y | 4.7 (14) | 4.4 (8) | 4.2 (16) | 4.6 (124) | 4.5 (162) |
| T69D | 2.7 (8) | 2.2 (4) | 2.6 (10) | 3.4 (92) | 3.2 (114) |
| T215C | 0 (0) | 0 (0) | 2.6 (10) | 3.5 (96) | 3 (106) |
| T215E | 0 (0) | 2.2 (4) | 2.1 (8) | 3.3 (89) | 2.8 (101) |
| K219E | 1.3 (4) | 2.2 (4) | 3.9 (15) | 1.7 (46) | 1.9 (69) |
| T215F | 3 (9) | 1.6 (3) | 2.6 (10) | 1.6 (43) | 1.8 (65) |
| D67G | 2 (6) | 1.6 (3) | 1.1 (4) | 1.3 (35) | 1.3 (48) |
| F77L | 1 (3) | 1.1 (2) | 1.8 (7) | 1.3 (35) | 1.3 (47) |
| L74V | 1.7 (5) | 1.6 (3) | 0.8 (3) | 1.2 (32) | 1.2 (43) |
| K219R | 1.3 (4) | 1.1 (2) | 1.8 (7) | 1.1 (29) | 1.2 (42) |
| V75M | 1.3 (4) | 8.2 (15) | 2.1 (8) | 0.5 (14) | 1.1 (41) |
| K65R | 2 (6) | 4.4 (8) | 0.5 (2) | 0.8 (23) | 1.1 (39) |
| K219N | 1.3 (4) | 2.7 (5) | 1.8 (7) | 0.8 (22) | 1.1 (38) |
| L74I | 1 (3) | 4.4 (8) | 0.5 (2) | 0.8 (22) | 1 (35) |
| T215I | 1.7 (5) | 1.1 (2) | 1.3 (5) | 0.7 (18) | 0.8 (30) |
| M184I | 1 (3) | 3.8 (7) | 0.3 (1) | 0.4 (12) | 0.6 (23) |
| F116Y | 0.7 (2) | 1.1 (2) | 0.3 (1) | 0.6 (16) | 0.6 (21) |
| T215V | 0 (0) | 0 (0) | 0.3 (1) | 0.7 (19) | 0.6 (20) |
| Y115F | 2 (6) | 0 (0) | 0 (0) | 0.5 (13) | 0.5 (19) |
| Q151M | 0 (0) | 1.6 (3) | 0.3 (1) | 0.6 (15) | 0.5 (19) |
| D67E | 2 (6) | 0.5 (1) | 0 (0) | 0.4 (10) | 0.5 (17) |
| K70E | 2.7 (8) | 0 (0) | 0 (0) | 0.2 (6) | 0.4 (14) |
| V75A | 1.3 (4) | 0.5 (1) | 0.3 (1) | 0.3 (7) | 0.4 (13) |
| V75S | 0.7 (2) | 0 (0) | 0 (0) | 0.1 (2) | 0.1 (4) |
| V75T | 0.7 (2) | 0 (0) | 0 (0) | 0.1 (2) | 0.1 (4) |
| T69ins | 0 (0) | 0 (0) | 0 (0) | 0 (0) | 0 (0) |
| ^a^The region “Latin America” includes three studies from Caribbean countries. The region “Upper-Income Country Regions” includes Europe, North America and upper-income Asian countries. “All Regions” includes pooled viruses with one or more NRTI SDRMs from all regions. SDRMs are shown in the order of the proportion in the “All Regions”; the number of NRTI SDRMs is indicated in each region (n). | | | | | |
