# Supplementary material for: Geographic and Temporal Trends in the Molecular Epidemiology and Genetic Mechanisms of Transmitted HIV-1 Drug Resistance: An Individual-Patient- and Sequence-Level Meta-Analysis
Source: PLoS Med. 2015 Apr 7;12(4):e1001810. doi: 10.1371/journal.pmed.1001810 (PMC4388826; doi:10.1371/journal.pmed.1001810)
Supplement: S5 Table — (DOCX) [file pmed.1001810.s008.docx]

| S5 Table. Proportion of each NNRTI SDRM According to Region*^a^* | | | | | |
| --- | --- | --- | --- | --- | --- |
| SDRM | Sub-Saharan Africa  (n=306)  % | South/ Southeast Asia  (n=136)  % | Latin America  (n=205)  % | Upper-Income Country Regions  (n=1,140)  % | All  Regions  (n=1,787)  % |
| K103N | 45 (137) | 28 (38) | 49 (100) | 54 (612) | 50 (887) |
| Y181C | 17 (53) | 32 (44) | 11 (23) | 12 (133) | 14 (253) |
| G190A | 14 (42) | 13 (18) | 7.8 (16) | 9.4 (107) | 10 (183) |
| K101E | 10 (32) | 7.4 (10) | 12 (25) | 5.1 (58) | 7 (125) |
| P225H | 1.6 (5) | 3.7 (5) | 3.4 (7) | 3.6 (41) | 3.3 (58) |
| Y188L | 1 (3) | 1.5 (2) | 3.4 (7) | 4 (45) | 3.2 (57) |
| K103S | 2 (6) | 2.9 (4) | 2 (4) | 1.7 (19) | 1.9 (33) |
| L100I | 2.3 (7) | 0 (0) | 2 (4) | 1.8 (21) | 1.8 (32) |
| G190S | 0.3 (1) | 1.5 (2) | 2.9 (6) | 1.2 (14) | 1.3 (23) |
| V106A | 0.7 (2) | 1.5 (2) | 0.5 (1) | 1.2 (14) | 1.1 (19) |
| V106M | 1 (3) | 3.7 (5) | 1 (2) | 0.8 (9) | 1.1 (19) |
| Y188H | 0.7 (2) | 0 (0) | 0 (0) | 1.4 (16) | 1 (18) |
| G190E | 1.3 (4) | 2.9 (4) | 0.5 (1) | 0.8 (9) | 1 (18) |
| Y188C | 1 (3) | 0.7 (1) | 0.5 (1) | 1.1 (12) | 1 (17) |
| M230L | 0.3 (1) | 0 (0) | 2.4 (5) | 0.7 (8) | 0.8 (14) |
| K101P | 0.3 (1) | 0 (0) | 1 (2) | 0.8 (9) | 0.7 (12) |
| Y181I | 0.3 (1) | 0.7 (1) | 0 (0) | 0.7 (8) | 0.6 (10) |
| V179F | 0.7 (2) | 0 (0) | 0 (0) | 0.4 (4) | 0.3 (6) |
| Y181V | 0.3 (1) | 0 (0) | 0.5 (1) | 0.1 (1) | 0.2 (3) |
| ^a^The region “Latin America” includes three studies from Caribbean countries. The region “Upper-Income Country Regions” includes Europe, North America and upper-income Asian countries. “All Regions” includes pooled viruses with one or more NNRTI SDRMs from all regions. SDRMs are shown in the order of the proportion in the “All Regions”; the number of NNRTI SDRMs is indicated in each region (n). | | | | | |
