# Supplementary material for: Geographic and Temporal Trends in the Molecular Epidemiology and Genetic Mechanisms of Transmitted HIV-1 Drug Resistance: An Individual-Patient- and Sequence-Level Meta-Analysis
Source: PLoS Med. 2015 Apr 7;12(4):e1001810. doi: 10.1371/journal.pmed.1001810 (PMC4388826; doi:10.1371/journal.pmed.1001810)
Supplement: S6 Table — (DOCX) [file pmed.1001810.s009.docx]

| S6 Table. Proportion of each PI SDRM According to Region*^a^* | | | | | |
| --- | --- | --- | --- | --- | --- |
| SDRM | Sub-Saharan Africa  (n=117)  % | South/ Southeast Asia  (n=79)  % | Latin America  (n=156)  % | Upper-Income Country Regions  (n=986)  % | All  Regions  (n=1,338)  % |
| L90M | 14 (16) | 8.9 (7) | 23 (36) | 19 (184) | 18 (243) |
| M46I | 14 (16) | 33 (26) | 12 (19) | 16 (159) | 16 (220) |
| M46L | 21 (24) | 18 (14) | 10 (16) | 12 (114) | 13 (168) |
| V82A | 0.9 (1) | 5.1 (4) | 9 (14) | 7.5 (74) | 7 (93) |
| I85V | 13 (15) | 6.3 (5) | 7.8 (12) | 6 (59) | 6.8 (91) |
| I54V | 3.4 (4) | 1.3 (1) | 6.4 (10) | 6.8 (67) | 6.1 (82) |
| N88D | 3.4 (4) | 3.8 (3) | 6.4 (10) | 4.9 (48) | 4.9 (65) |
| I84V | 1.7 (2) | 3.8 (3) | 3.9 (6) | 4.8 (47) | 4.3 (58) |
| D30N | 1.7 (2) | 1.3 (1) | 5.1 (8) | 4.4 (43) | 4 (54) |
| G73S | 3.4 (4) | 2.5 (2) | 1.3 (2) | 2 (20) | 2.1 (28) |
| F53L | 1.7 (2) | 0 (0) | 0.6 (1) | 2.3 (23) | 1.9 (26) |
| L24I | 1.7 (2) | 1.3 (1) | 0.6 (1) | 2.1 (21) | 1.9 (25) |
| I54L | 0.9 (1) | 0 (0) | 0.6 (1) | 1.8 (18) | 1.5 (20) |
| V32I | 0 (0) | 1.3 (1) | 1.3 (2) | 1.5 (15) | 1.4 (18) |
| I50V | 1.7 (2) | 1.3 (1) | 1.3 (2) | 1.3 (13) | 1.4 (18) |
| I47V | 0.9 (1) | 0 (0) | 0.6 (1) | 1.5 (15) | 1.3 (17) |
| L23I | 2.6 (3) | 1.3 (1) | 0 (0) | 0.8 (8) | 0.9 (12) |
| F53Y | 4.3 (5) | 3.8 (3) | 0 (0) | 0.3 (3) | 0.8 (11) |
| V82T | 0.9 (1) | 0 (0) | 1.9 (3) | 0.7 (7) | 0.8 (11) |
| N88S | 0.9 (1) | 0 (0) | 0.6 (1) | 0.9 (9) | 0.8 (11) |
| L76V | 1.7 (2) | 0 (0) | 1.3 (2) | 0.4 (4) | 0.6 (8) |
| V82L | 0 (0) | 2.5 (2) | 0.6 (1) | 0.5 (5) | 0.6 (8) |
| N83D | 2.6 (3) | 2.5 (2) | 0.6 (1) | 0.2 (2) | 0.6 (8) |
| G48V | 0 (0) | 0 (0) | 1.3 (2) | 0.5 (5) | 0.5 (7) |
| I54T | 1.7 (2) | 0 (0) | 1.9 (3) | 0.1 (1) | 0.5 (6) |
| I50L | 1.7 (2) | 1.3 (1) | 0 (0) | 0.2 (2) | 0.4 (5) |
| G73A | 0.9 (1) | 0 (0) | 0 (0) | 0.4 (4) | 0.4 (5) |
| G73C | 0 (0) | 0 (0) | 0.6 (1) | 0.4 (4) | 0.4 (5) |
| V82F | 0.9 (1) | 1.3 (1) | 0 (0) | 0.2 (2) | 0.3 (4) |
| I54M | 0 (0) | 0 (0) | 0 (0) | 0.3 (3) | 0.2 (3) |
| G73T | 0 (0) | 0 (0) | 0 (0) | 0.3 (3) | 0.2 (3) |
| I54S | 0 (0) | 0 (0) | 0.6 (1) | 0.1 (1) | 0.2 (2) |
| V82S | 0 (0) | 0 (0) | 0 (0) | 0.2 (2) | 0.2 (2) |
| I54A | 0 (0) | 0 (0) | 0 (0) | 0.1 (1) | 0.1 (1) |
| I47A | 0 (0) | 0 (0) | 0 (0) | 0 (0) | 0 (0) |
| G48M | 0 (0) | 0 (0) | 0 (0) | 0 (0) | 0 (0) |
| V82C | 0 (0) | 0 (0) | 0 (0) | 0 (0) | 0 (0) |
| V82M | 0 (0) | 0 (0) | 0 (0) | 0 (0) | 0 (0) |
| I84A | 0 (0) | 0 (0) | 0 (0) | 0 (0) | 0 (0) |
| I84C | 0 (0) | 0 (0) | 0 (0) | 0 (0) | 0 (0) |
| ^a^The region “Latin America” includes three studies from Caribbean countries. The region “Upper-Income Country Regions” includes Europe, North America and upper-income Asian countries. “All Regions” includes pooled viruses with one or more PI SDRMs from all regions. SDRMs are shown in the order of the proportion in the “All Regions”; the number of PI SDRMs is indicated in each region (n). | | | | | |
