# Supplementary material for: Geographic and Temporal Trends in the Molecular Epidemiology and Genetic Mechanisms of Transmitted HIV-1 Drug Resistance: An Individual-Patient- and Sequence-Level Meta-Analysis
Source: PLoS Med. 2015 Apr 7;12(4):e1001810. doi: 10.1371/journal.pmed.1001810 (PMC4388826; doi:10.1371/journal.pmed.1001810)
Supplement: S7 Table — (DOCX) [file pmed.1001810.s010.docx]

| S7 Table. Proportion of each NRTI SDRM According to Subtype*^a^* | | | | | | | | |
| --- | --- | --- | --- | --- | --- | --- | --- | --- |
| SDRM | A  (n=126)  % | B  (n=2,920)  % | C  (n=133)  % | D  (n=96)  % | G  (n=50)  % | CRF01_AE  (n=157)  % | CRF02_AG  (n=72)  % | All Subtypes  (n=3,554)  % |
| M41L | 7.9 (10) | 18 (512) | 14 (18) | 24 (23) | 20 (10) | 6.4 (10) | 15 (11) | 17 (594) |
| M184V | 21 (27) | 9.2 (268) | 21 (28) | 20 (19) | 18 (9) | 21 (33) | 11 (8) | 11 (392) |
| D67N | 4.8 (6) | 8.4 (246) | 5.3 (7) | 8.3 (8) | 6 (3) | 7.6 (12) | 14 (10) | 8.2 (292) |
| T215S | 6.4 (8) | 8.4 (246) | 3.8 (5) | 0 (0) | 0 (0) | 3.8 (6) | 8.3 (6) | 7.6 (271) |
| T215D | 2.4 (3) | 8.5 (247) | 0.8 (1) | 1 (1) | 2 (1) | 0.6 (1) | 0 (0) | 7.2 (254) |
| L210W | 3.2 (4) | 7 (204) | 4.5 (6) | 8.3 (8) | 6 (3) | 6.4 (10) | 2.8 (2) | 6.7 (237) |
| K219Q | 8.7 (11) | 6.4 (187) | 2.3 (3) | 3.1 (3) | 6 (3) | 6.4 (10) | 6.9 (5) | 6.3 (222) |
| K70R | 4 (5) | 4.6 (134) | 3.8 (5) | 7.3 (7) | 4 (2) | 4.5 (7) | 5.6 (4) | 4.6 (164) |
| T215Y | 4 (5) | 4.5 (131) | 3.8 (5) | 10 (10) | 6 (3) | 3.8 (6) | 2.8 (2) | 4.6 (162) |
| T69D | 0.8 (1) | 3.5 (101) | 4.5 (6) | 3.1 (3) | 0 (0) | 1.3 (2) | 0 (0) | 3.2 (113) |
| T215C | 6.4 (8) | 3.3 (96) | 1.5 (2) | 1 (1) | 0 (0) | 0 (0) | 0 (0) | 3 (107) |
| T215E | 0 (0) | 3.3 (97) | 0 (0) | 0 (0) | 4 (2) | 0 (0) | 1.4 (1) | 2.8 (100) |
| K219E | 0 (0) | 2 (57) | 2.3 (3) | 1 (1) | 4 (2) | 1.9 (3) | 4.2 (3) | 1.9 (69) |
| T215F | 4.8 (6) | 1.5 (44) | 2.3 (3) | 2.1 (2) | 4 (2) | 1.9 (3) | 1.4 (1) | 1.7 (61) |
| D67G | 1.6 (2) | 1.3 (37) | 3.8 (5) | 2.1 (2) | 0 (0) | 1.9 (3) | 0 (0) | 1.4 (49) |
| F77L | 3.2 (4) | 1.3 (39) | 0.8 (1) | 1 (1) | 0 (0) | 1.3 (2) | 1.4 (1) | 1.4 (48) |
| K219R | 4 (5) | 1 (29) | 4.5 (6) | 0 (0) | 2 (1) | 1.9 (3) | 1.4 (1) | 1.3 (45) |
| V75M | 2.4 (3) | 0.7 (21) | 0 (0) | 0 (0) | 2 (1) | 10.2 (16) | 0 (0) | 1.2 (41) |
| L74V | 0.8 (1) | 1.1 (32) | 2.3 (3) | 0 (0) | 2 (1) | 1.3 (2) | 1.4 (1) | 1.1 (40) |
| K65R | 2.4 (3) | 0.7 (21) | 3 (4) | 0 (0) | 4 (2) | 4.5 (7) | 0 (0) | 1 (37) |
| K219N | 1.6 (2) | 0.8 (24) | 2.3 (3) | 2.1 (2) | 2 (1) | 2.6 (4) | 1.4 (1) | 1 (37) |
| L74I | 2.4 (3) | 0.7 (20) | 0.8 (1) | 1 (1) | 2 (1) | 3.8 (6) | 2.8 (2) | 1 (34) |
| T215I | 1.6 (2) | 0.7 (21) | 1.5 (2) | 1 (1) | 0 (0) | 1.3 (2) | 2.8 (2) | 0.8 (30) |
| M184I | 0.8 (1) | 0.5 (15) | 3 (4) | 0 (0) | 0 (0) | 1.9 (3) | 1.4 (1) | 0.7 (24) |
| F116Y | 0.8 (1) | 0.6 (16) | 1.5 (2) | 0 (0) | 0 (0) | 0.6 (1) | 1.4 (1) | 0.6 (21) |
| Q151M | 0 (0) | 0.6 (17) | 0 (0) | 0 (0) | 2 (1) | 1.3 (2) | 0 (0) | 0.6 (20) |
| T215V | 0.8 (1) | 0.6 (18) | 0 (0) | 0 (0) | 2 (1) | 0 (0) | 0 (0) | 0.6 (20) |
| D67E | 1.6 (2) | 0.3 (10) | 2.3 (3) | 1 (1) | 0 (0) | 0.6 (1) | 2.8 (2) | 0.5 (19) |
| Y115F | 0.8 (1) | 0.4 (12) | 0 (0) | 1 (1) | 2 (1) | 0 (0) | 5.6 (4) | 0.5 (19) |
| K70E | 0 (0) | 0.2 (7) | 1.5 (2) | 1 (1) | 0 (0) | 0.6 (1) | 4.2 (3) | 0.4 (14) |
| V75A | 0.8 (1) | 0.2 (7) | 2.3 (3) | 0 (0) | 0 (0) | 0.6 (1) | 0 (0) | 0.3 (12) |
| V75S | 0 (0) | 0.1 (2) | 0.8 (1) | 0 (0) | 0 (0) | 0 (0) | 0 (0) | 0.1 (3) |
| V75T | 0 (0) | 0.1 (2) | 0.8 (1) | 0 (0) | 0 (0) | 0 (0) | 0 (0) | 0.1 (3) |
| T69ins | 0 (0) | 0 (0) | 0 (0) | 0 (0) | 0 (0) | 0 (0) | 0 (0) | 0 (0) |
| ^a^The region “All Subtypes” includes pooled viruses with one or more NRTI SDRMs from all subtypes. SDRMs are shown in the order of the proportion in the “All Subtypes”; the number of NRTI SDRMs is indicated in each subtype (n). | | | | | | | | |
