# Supplementary material for: Geographic and Temporal Trends in the Molecular Epidemiology and Genetic Mechanisms of Transmitted HIV-1 Drug Resistance: An Individual-Patient- and Sequence-Level Meta-Analysis
Source: PLoS Med. 2015 Apr 7;12(4):e1001810. doi: 10.1371/journal.pmed.1001810 (PMC4388826; doi:10.1371/journal.pmed.1001810)
Supplement: S8 Table — (DOCX) [file pmed.1001810.s011.docx]

| S8 Table. Proportion of each NNRTI SDRM According to Subtype*^a^* | | | | | | | | |
| --- | --- | --- | --- | --- | --- | --- | --- | --- |
| SDRM | A  (n=82)  % | B  (n=1,212)  % | C  (n=179)  % | D  (n=61)  % | G  (n=32)  % | CRF01_AE  (n=115)  % | CRF02_AG  (n=65)  % | All  Subtypes  (n=1,746)  % |
| K103N | 50 (41) | 53 (648) | 42 (75) | 36 (22) | 41 (13) | 30 (34) | 45 (29) | 49 (862) |
| Y181C | 26 (21) | 11 (133) | 19 (34) | 15 (9) | 19 (6) | 33 (38) | 14 (9) | 14 (250) |
| G190A | 8.5 (7) | 9.4 (114) | 13 (24) | 21 (13) | 6.3 (2) | 13 (15) | 11 (7) | 10 (182) |
| K101E | 1.2 (1) | 6 (73) | 11 (19) | 15 (9) | 16 (5) | 5.2 (6) | 9.2 (6) | 6.8 (119) |
| Y188L | 1.2 (1) | 4.2 (51) | 1.1 (2) | 0 (0) | 13 (4) | 0 (0) | 0 (0) | 3.3 (58) |
| P225H | 2.4 (2) | 3.2 (39) | 0.6 (1) | 1.6 (1) | 3 (1) | 3.5 (4) | 14 (9) | 3.3 (57) |
| K103S | 1.2 (1) | 2.1 (25) | 2.8 (5) | 1.6 (1) | 0 (0) | 0.9 (1) | 0 (0) | 1.9 (33) |
| L100I | 1.2 (1) | 2.1 (25) | 1.1 (2) | 4.9 (3) | 0 (0) | 0.9 (1) | 0 (0) | 1.8 (32) |
| G190S | 0 (0) | 1.4 (17) | 0.6 (1) | 0 (0) | 0 (0) | 2.6 (3) | 3.1 (2) | 1.3 (23) |
| V106M | 0 (0) | 0.6 (7) | 4.5 (8) | 0 (0) | 0 (0) | 2.6 (3) | 0 (0) | 1 (18) |
| Y188H | 0 (0) | 1.2 (15) | 0 (0) | 3.3 (2) | 0 (0) | 0 (0) | 1.5 (1) | 1 (18) |
| G190E | 0 (0) | 0.7 (9) | 0.6 (1) | 1.6 (1) | 0 (0) | 4.4 (5) | 3.1 (2) | 1 (18) |
| V106A | 1.2 (1) | 1.2 (15) | 0 (0) | 0 (0) | 0 (0) | 0.9 (1) | 0 (0) | 1 (17) |
| Y188C | 2.4 (2) | 0.8 (10) | 0.6 (1) | 0 (0) | 3 (1) | 1.7 (2) | 0 (0) | 0.9 (16) |
| M230L | 1.2 (1) | 0.9 (11) | 0.6 (1) | 0 (0) | 0 (0) | 0.9 (1) | 0 (0) | 0.8 (14) |
| K101P | 1.2 (1) | 0.8 (10) | 0.6 (1) | 0 (0) | 0 (0) | 0 (0) | 0 (0) | 0.7 (12) |
| Y181I | 1.2 (1) | 0.4 (5) | 2.2 (4) | 0 (0) | 0 (0) | 0.9 (1) | 0 (0) | 0.6 (11) |
| V179F | 0 (0) | 0.3 (3) | 0 (0) | 0 (0) | 0 (0) | 0 (0) | 0 (0) | 0.2 (3) |
| Y181V | 1.2 (1) | 0.2 (2) | 0 (0) | 0 (0) | 0 (0) | 0 (0) | 0 (0) | 0.2 (3) |
| ^a^The region “All Subtypes” includes pooled viruses with one or more NNRTI SDRMs from all subtypes. SDRMs are shown in the order of the proportion in the “All Subtypes”; the number of NNRTI SDRMs is indicated in each subtype (n). | | | | | | | | |
