# Supplementary material for: Geographic and Temporal Trends in the Molecular Epidemiology and Genetic Mechanisms of Transmitted HIV-1 Drug Resistance: An Individual-Patient- and Sequence-Level Meta-Analysis
Source: PLoS Med. 2015 Apr 7;12(4):e1001810. doi: 10.1371/journal.pmed.1001810 (PMC4388826; doi:10.1371/journal.pmed.1001810)
Supplement: S9 Table — (DOCX) [file pmed.1001810.s012.docx]

| S9 Table. Proportion of each PI SDRM According to Subtype*^a^* | | | | | | | | |
| --- | --- | --- | --- | --- | --- | --- | --- | --- |
| SDRM | A  (n=51)  % | B  (n=1,039)  % | C  (n=79)  % | D  (n=26)  % | G  (n=15)  % | CRF01_AE  (n=71)  % | CRF02_AG  (n=37)  % | All  Subtypes (n=1,318)  % |
| L90M | 5.9 (3) | 20 (207) | 15 (12) | 12 (3) | 27 (4) | 8.5 (6) | 19 (7) | 18 (242) |
| M46I | 16 (8) | 16 (163) | 24 (19) | 0 (0) | 13 (2) | 28 (20) | 24 (9) | 17 (221) |
| M46L | 18 (9) | 11 (117) | 14 (11) | 15 (4) | 6.7 (1) | 25 (18) | 8.1 (3) | 12 (163) |
| I85V | 5.9 (3) | 6.7 (70) | 13 (10) | 23 (6) | 0 (0) | 4.2 (3) | 2.7 (1) | 7.1 (93) |
| V82A | 0 (0) | 7.3 (76) | 3.8 (3) | 12 (3) | 0 (0) | 2.8 (2) | 2.7 (1) | 6.5 (85) |
| I54V | 3.9 (2) | 6.3 (65) | 2.5 (2) | 3.9 (1) | 13 (2) | 1.4 (1) | 5.4 (2) | 5.7 (75) |
| N88D | 3.9 (2) | 5.4 (56) | 3.8 (3) | 7.7 (2) | 0 (0) | 2.8 (2) | 0 (0) | 4.9 (65) |
| I84V | 2 (1) | 4.5 (47) | 2.5 (2) | 0 (0) | 13 (2) | 5.6 (4) | 5.4 (2) | 4.4 (58) |
| D30N | 2 (1) | 4.9 (51) | 1.3 (1) | 3.9 (1) | 0 (0) | 0 (0) | 0 (0) | 4.1 (54) |
| G73S | 2 (1) | 2.2 (23) | 3.8 (3) | 0 (0) | 0 (0) | 0 (0) | 0 (0) | 2.1 (27) |
| F53L | 2 (1) | 1.8 (19) | 5.1 (4) | 3.9 (1) | 0 (0) | 1.4 (1) | 0 (0) | 2 (26) |
| L24I | 2 (1) | 1.7 (18) | 0 (0) | 3.9 (1) | 6.7 (1) | 1.4 (1) | 2.7 (1) | 1.8 (23) |
| I54L | 3.9 (2) | 1.5 (16) | 1.3 (1) | 0 (0) | 0 (0) | 0 (0) | 2.7 (1) | 1.5 (20) |
| L23I | 16 (8) | 0.7 (7) | 2.5 (2) | 3.9 (1) | 0 (0) | 1.4 (1) | 0 (0) | 1.4 (19) |
| V32I | 2 (1) | 1.4 (15) | 0 (0) | 3.9 (1) | 0 (0) | 1.4 (1) | 0 (0) | 1.4 (18) |
| I47V | 3.9 (2) | 1.2 (12) | 0 (0) | 0 (0) | 6.7 (1) | 0 (0) | 2.7 (1) | 1.2 (16) |
| I50V | 0 (0) | 1.3 (13) | 0 (0) | 3.9 (1) | 0 (0) | 1.4 (1) | 0 (0) | 1.1 (15) |
| F53Y | 0 (0) | 0.3 (3) | 1.3 (1) | 0 (0) | 0 (0) | 4.2 (3) | 11 (4) | 0.8 (11) |
| V82T | 0 (0) | 0.8 (8) | 1.3 (1) | 0 (0) | 13 (2) | 0 (0) | 0 (0) | 0.8 (11) |
| N88S | 2 (1) | 0.9 (9) | 0 (0) | 0 (0) | 0 (0) | 0 (0) | 2.7 (1) | 0.8 (11) |
| N83D | 2 (1) | 0.2 (2) | 1.3 (1) | 0 (0) | 0 (0) | 4.2 (3) | 2.7 (1) | 0.6 (8) |
| G48V | 0 (0) | 0.7 (7) | 0 (0) | 0 (0) | 0 (0) | 0 (0) | 0 (0) | 0.5 (7) |
| I54T | 5.9 (3) | 0.3 (3) | 0 (0) | 0 (0) | 0 (0) | 0 (0) | 2.7 (1) | 0.5 (7) |
| V82L | 0 (0) | 0.6 (6) | 0 (0) | 0 (0) | 0 (0) | 1.4 (1) | 0 (0) | 0.5 (7) |
| L76V | 0 (0) | 0.4 (4) | 1.3 (1) | 3.9 (1) | 0 (0) | 0 (0) | 0 (0) | 0.5 (6) |
| I50L | 0 (0) | 0.2 (2) | 2.5 (2) | 0 (0) | 0 (0) | 1.4 (1) | 0 (0) | 0.4 (5) |
| G73A | 2 (1) | 0.3 (3) | 0 (0) | 0 (0) | 0 (0) | 0 (0) | 2.7 (1) | 0.4 (5) |
| G73C | 0 (0) | 0.5 (5) | 0 (0) | 0 (0) | 0 (0) | 0 (0) | 0 (0) | 0.4 (5) |
| V82F | 0 (0) | 0.2 (2) | 0 (0) | 0 (0) | 0 (0) | 1.4 (1) | 2.7 (1) | 0.3 (4) |
| I54M | 0 (0) | 0.3 (3) | 0 (0) | 0 (0) | 0 (0) | 0 (0) | 0 (0) | 0.2 (3) |
| G73T | 0 (0) | 0.3 (3) | 0 (0) | 0 (0) | 0 (0) | 0 (0) | 0 (0) | 0.2 (3) |
| I54S | 0 (0) | 0.2 (2) | 0 (0) | 0 (0) | 0 (0) | 0 (0) | 0 (0) | 0.2 (2) |
| V82S | 0 (0) | 0.1 (1) | 0 (0) | 0 (0) | 0 (0) | 1.4 (1) | 0 (0) | 0.2 (2) |
| I54A | 0 (0) | 0.1 (1) | 0 (0) | 0 (0) | 0 (0) | 0 (0) | 0 (0) | 0.1 (1) |
| I47A | 0 (0) | 0 (0) | 0 (0) | 0 (0) | 0 (0) | 0 (0) | 0 (0) | 0 (0) |
| G48M | 0 (0) | 0 (0) | 0 (0) | 0 (0) | 0 (0) | 0 (0) | 0 (0) | 0 (0) |
| V82C | 0 (0) | 0 (0) | 0 (0) | 0 (0) | 0 (0) | 0 (0) | 0 (0) | 0 (0) |
| V82M | 0 (0) | 0 (0) | 0 (0) | 0 (0) | 0 (0) | 0 (0) | 0 (0) | 0 (0) |
| I84A | 0 (0) | 0 (0) | 0 (0) | 0 (0) | 0 (0) | 0 (0) | 0 (0) | 0 (0) |
| I84C | 0 (0) | 0 (0) | 0 (0) | 0 (0) | 0 (0) | 0 (0) | 0 (0) | 0 (0) |
| ^a^The region “All Subtypes” includes pooled viruses with one or more PI SDRMs from all subtypes. SDRMs are shown in the order of the proportion in the “All Subtypes”; the number of PI SDRMs is indicated in each subtype (n). | | | | | | | | |
